# Supplementary material for: Transcriptomic analysis of the immune response to in vivo gene electrotransfer in colorectal cancer
Source: Mol Ther Nucleic Acids. 2025 Jan 16;36(1):102448. doi: 10.1016/j.omtn.2025.102448 (PMC11834060; doi:10.1016/j.omtn.2025.102448)
Supplement: Document S1. Figures S1–S3 and Tables S1–S4 [file mmc1.pdf]

OMTN, Volume *36*

Supplemental information

Transcriptomic analysis of the immune response to *in vivo* gene electrotransfer in colorectal cancer

Mariangela De Robertis, Tim Bozic, Iva Santek, Flaviana Marzano, Bostjan Markelc, Domenico Alessandro Silvestris, Apollonia Tullo, Graziano Pesole, Maja Cemazar, and Emanuela Signori

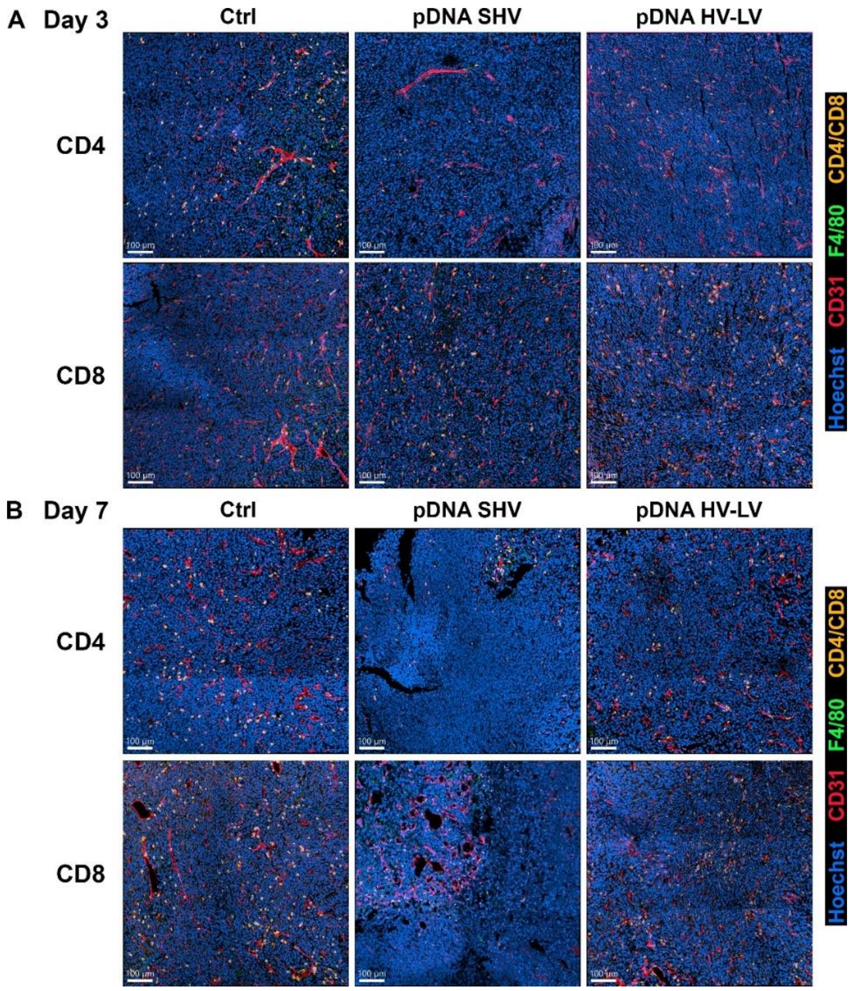

Figure S1. Immune cell infiltration in the tumor centers on day three and day seven after pDNA GET in CT26 murine tumors using SHV or HV-LV pulse protocols. GET was performed using SHV or HV-LV pulses. Tumors were collected on **A)** day three and **B)** day seven after GET for histological analysis. Frozen sections of tumor tissue were stained with anti-CD4 or anti-CD8 (yellow, Cy3), anti-CD31 (red, Alexa 647), anti-F4/80 (green, Alexa 488) and Hoechst 33342 (blue). The representative images of tumor centers are shown. Yellow: Cy3; Red: Alexa 647; Green: Alexa 488; Blue: Hoechst 33342. Scale bar: 100 mm.

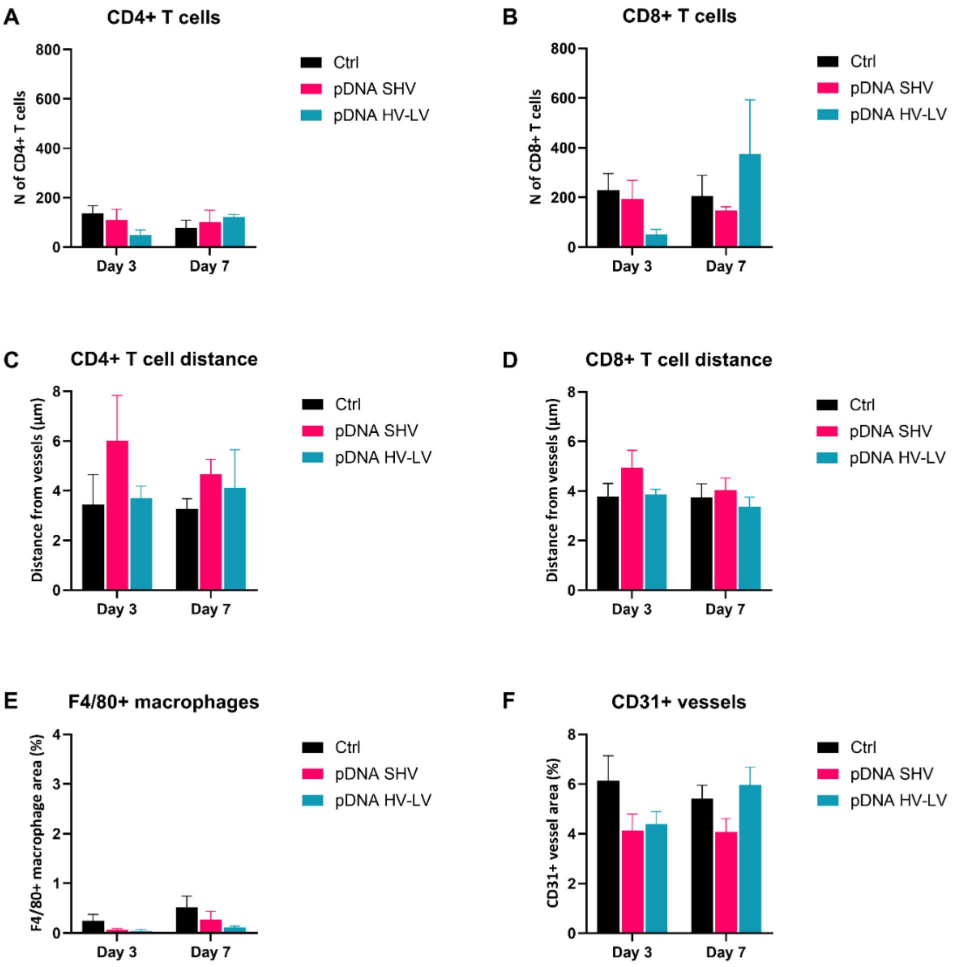

Figure S2. Quantification of immunofluorescence data obtained from images of CT26 tumor centers showed negligible changes in the tumor microenvironment. Graphs represent number of **A)** CD4, **B)** CD8 positive T cells and their **C, D)** distance to tumor vessels, **E)** F4/80 positive macrophages and **F)** the percentage of tumor vessels in CT26 tumors on day 3 and day 7 after GET of pDNA using SHV or HV-LV pulse protocol. Data are presented as AM ± SEM (n ≥ 5). Statistical significance was determined by One-way ANOVA.

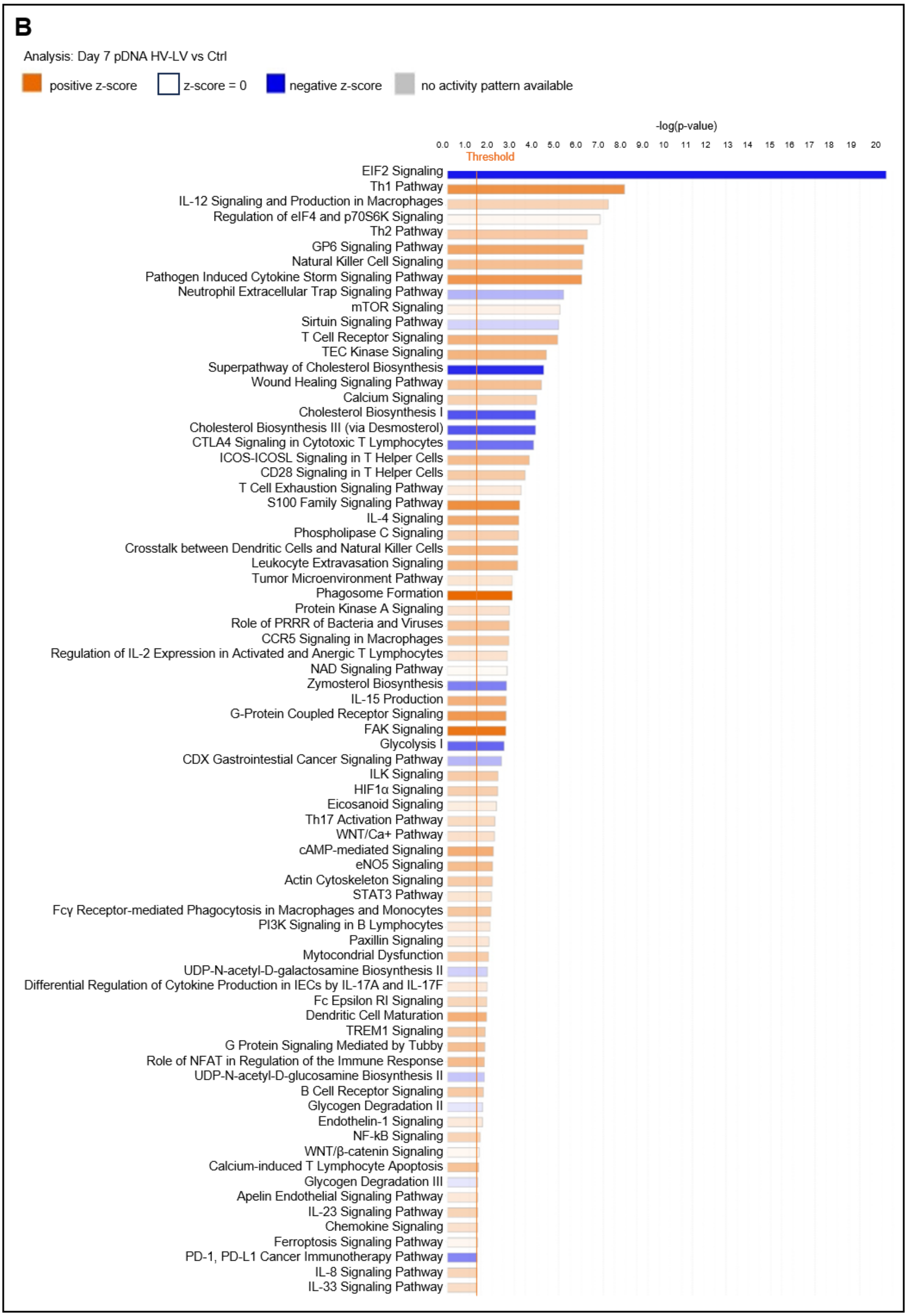

Figure S3. Complete list of the enriched GO canonical pathways in pDNA HV-LV group. Analysis of tumors treated with pDNA GET using H-LV pulse protocol compared to controls at seven days. The plot shows the GO molecular function terms plotted in order of significance.

Table S1. Electric parameters of SHV and HV-LV pulse protocols used for GET of pDNA to CT26 tumors.

Pulse protocol	SHV	HV-LV	
N of pulses	8	4x (1HV + 1LV)	
Electrode distance	6 mm	6 mm	
Voltage/distance [V/cm]	1300	HV: 1300	LV: 150
Duration [ms]	0.1	HV: 0.1	LV: 20
Frequency [Hz]	1	1	
Time between HV and LV [ms]	/	50	
Time between (1HV + 1 LV) [s]	/	1	

Table S2. List of primary and secondary antibodies used for immunofluorescence.

**Primary antibodies**	**Supplier**	**Cat. No.**	**Dilution**
Anti-CD4 antibody	Abcam	ab183685	1:200
Anti-CD8 antibody	Abcam	ab209775	1:200
Mouse/Rat CD31/PECAM-1 Antibody	R&D systems	AF3628	1:200
F4/80 Monoclonal Antibody (BM8)	ThermoFisher Scientific	14-4801-82	1:200
Secondary antibodies	**Supplier**	**Cat. No.**	**Dilution**
Donkey Anti-Rabbit IgG Antibody (Cy3®)	Jackson Immunoresearch	711-165-152	1:400
Donkey Anti-Goat IgG Antibody (Alexa Fluor® 647)	Jackson Immunoresearch	705-605-147	1:400
Donkey Anti-Rat IgG (Alexa Fluor® 488)	Jackson Immunoresearch	712-545-150	1:400

Table S3. Statistical analysis of the number of CD8+T cells at the tumor edges at day three and day seven after pDNA GET in CT26 murine tumors using SHV or HV-LV pulse protocols.

Ordinary one-way ANOVA (Holm-Šídák)	Mean Diff.	Below threshold?	Summary	Adj. P Value[§]
Ctrl d3 vs. pDNA Ctrl (SHV) d7	-376.7	Yes	*	0.0438
pDNA Ctrl (SHV) d3 vs. pDNA Ctrl (SHV) d7	-434.5	Yes	*	0.0109
pDNA Ctrl (HV-LV) d3 vs. pDNA Ctrl (SHV) d7	-543.8	Yes	*	0.0109

[§]Statistical significance was determined by One-way ANOVA. Adj. P-value of < 0.05 was considered to be statistically significant (*P < 0.05 vs non-treated tumors (Ctrl)), between treatments or time points.

Table S4. Statistical analysis of the number of F4/80+ macrophages at the tumor edges at day three and day seven after pDNA GET in CT26 murine tumors using SHV or HV-LV pulse protocols.

Ordinary one-way ANOVA (Holm-Šídák)	Mean Diff.	Below threshold?	Summary	Adj. P Value[§]
Ctrl d3 vs. Ctrl d7	1.465	Yes	***	0.0010
Ctrl d3 vs. pDNA Ctrl (SHV) d3	1.267	Yes	*	0.0159
Ctrl d3 vs. pDNA Ctrl (HV-LV) d7	1.304	Yes	*	0.0136
Ctrl d7 vs. pDNA Ctrl (SHV) d7	-1.029	Yes	**	0.0047
Ctrl d7 vs. pDNA Ctrl (HV-LV) d3	-2.474	Yes	****	<0.0001
pDNA Ctrl (SHV) d3 vs. pDNA Ctrl (HV-LV) d3	-2.275	Yes	****	<0.0001
pDNA Ctrl (SHV) d7 vs. pDNA Ctrl (HV-LV) d3	-1.444	Yes	**	0.0015
pDNA Ctrl (HV-LV) d3 vs. pDNA Ctrl (HV-LV) d7	2.312	Yes	****	<0.0001

[§]Statistical significance was determined by One-way ANOVA. Adj. P-value of < 0.05 was considered to be statistically significant (*P < 0.05, **P < 0.01, ****P < 0.0001 vs non-treated tumors (Ctrl)), between treatments or time points.
