# Supplementary material for: Transcriptomic analysis of the immune response to in vivo gene electrotransfer in colorectal cancer
Source: Mol Ther Nucleic Acids. 2025 Jan 16;36(1):102448. doi: 10.1016/j.omtn.2025.102448 (PMC11834060; doi:10.1016/j.omtn.2025.102448)
Supplement: Document S2. Article plus supplemental information [file mmc2.pdf]

Molecular Therapy
Nucleic Acids
Original Article

Transcriptomic analysis of the immune response to *in vivo* gene electrotransfer in colorectal cancer

Mariangela De Robertis,[1,2,8] Tim Bozic,[3,8] Iva Santek,[3,4] Flaviana Marzano,[2] Bostjan Markelc,[3,5] Domenico Alessandro Silvestris,[1] Apollonia Tullo,[2] Graziano Pesole,[1,2] Maja Cemazar,[3,6] and Emanuela Signori[7]

[1]Department of Biosciences, Biotechnology, and Environment, University of Bari "Aldo Moro", 70126 Bari, Italy; [2]Institute of Biomembranes, Bioenergetics, and Molecular Biotechnologies, Consiglio Nazionale delle Ricerche, 70126 Bari, Italy; [3]Department of Experimental Oncology, Institute of Oncology Ljubljana, Zaloska cesta 2, 1000 Ljubljana, Slovenia; [4]Faculty of Medicine, University of Ljubljana, Vrazov trg 2, 1000 Ljubljana, Slovenia; [5]Biotechnical Faculty, University of Ljubljana, Jamnikarjeva ulica 101, 1000 Ljubljana, Slovenia; [6]Faculty of Health Sciences, University of Primorska, Polje 42, 6310 Izola, Slovenia; [7]Laboratory of Molecular Pathology and Experimental Oncology, Institute of Translational Pharmacology, Consiglio Nazionale delle Ricerche, 0133 Rome, Italy

Gene electrotransfer (GET) has recently emerged as an effective nonviral approach for plasmid DNA (pDNA) delivery in gene therapy for several pathologies, including cancer. Multiple mechanisms have been identified that influence cell biology after GET, as electroporation significantly increases pDNA uptake and immunogenicity, which may directly influence target cell death. However, the molecular effects of *in vivo* electroporation-mediated DNA delivery have yet to be fully elucidated. In this study, we evaluated the transcriptomes of murine colorectal tumors treated with two protocols, short- and high-voltage (SHV) electric pulses or an adapted high-voltage-low-voltage (HV-LV) pulse protocol, both of which are used for reversible electroporation. Although no significant differences in clinical outcomes were observed, variations in intratumoral macrophage infiltration were reported between the two treatment methods. Transcriptomic analysis revealed that apoptosis is a predominant mode of cell death after GET by SHV pulses, whereas GET by HV-LV pulses is associated with immunogenic necrotic pathways as well as the activation of both the innate and adaptive immune response. We demonstrated that specific pulse parameters can induce distinct immunomodulatory profiles in the tumor microenvironment; therefore, these aspects should be considered carefully when selecting the most suitable GET-based approach for antitumor immunization.

INTRODUCTION

Gene electrotransfer (GET) is a promising nonviral method for gene delivery in which plasmid DNA (pDNA) is transferred into various cells via electric pulses.[1] Compared with viral gene delivery, GET is a safer and more cost-effective method, and the application of electric pulses enhances the otherwise low transfection efficiency of pDNA in tissues.[2] This technique has shown great potential for treating various diseases, including infectious diseases, genetic disorders, and cancer.[3] It is an excellent tool for the delivery of therapeutic genes[4] and vaccines.[5] GET offers several advantages over viral vectors, such as safety, ease of use, low cost, and the ability to transfer large plasmids into various tissue types, including solid tumors.[6] Preclinical animal studies of intramuscular or intratumoral electroporation (EP) of pDNA encoding tumor antigens and/or immunostimulatory molecules[7,8] predict that GET-based approaches could be translated for use in veterinary and clinical therapeutic protocols. Currently, this technique is used in veterinary practice for immunotherapy.[9] Recently, the combination of GET and electrochemotherapy (ECT) has been explored.[10] GET protocols for plasmid delivery have also been tested in several clinical trials involving both intramuscular[11–13] and intratumoral immunotherapy,[14–18] supporting the safety and potential impact of pDNA EP.

Currently, a very active area of research involves improving pDNA electrotransfer protocols to minimize tissue damage and increase gene transfection efficiency[19,20]; however, the molecular effects of DNA delivery *in vivo* remain poorly characterized.

Recent advancements in omics technologies have greatly expanded the potential for analyzing and understanding therapeutic mechanisms at the molecular level. An early RNA sequencing (RNA-seq) study in which mouse muscles were injected with pDNA and electroporated provided information on the molecular changes in immune signaling pathways in response to the intramuscular plasmid EP.[21,22] Most recently, RNA-seq was used to identify genes that

Received 20 June 2024; accepted 10 January 2025;
https://doi.org/10.1016/j.omtn.2025.102448.

[8]These authors contributed equally

Correspondence: Maja Cemazar, Department of Experimental Oncology, Institute of Oncology Ljubljana, Zaloska cesta 2, 1000 Ljubljana, Slovenia.
E-mail: mcemazar@onko-i.si
Correspondence: Emanuela Signori, Laboratory of Molecular Pathology and Experimental Oncology, Institute of Translational Pharmacology, Consiglio Nazionale delle Ricerche, Via Fosso del Cavaliere 100, 0133 Rome, Italy.
E-mail: emanuela.signori@ift.cnr.it

Published by Elsevier Inc. on behalf of The American Society of Gene and Cell Therapy.
This is an open access article under the CC BY license (http://creativecommons.org/licenses/by/4.0/).

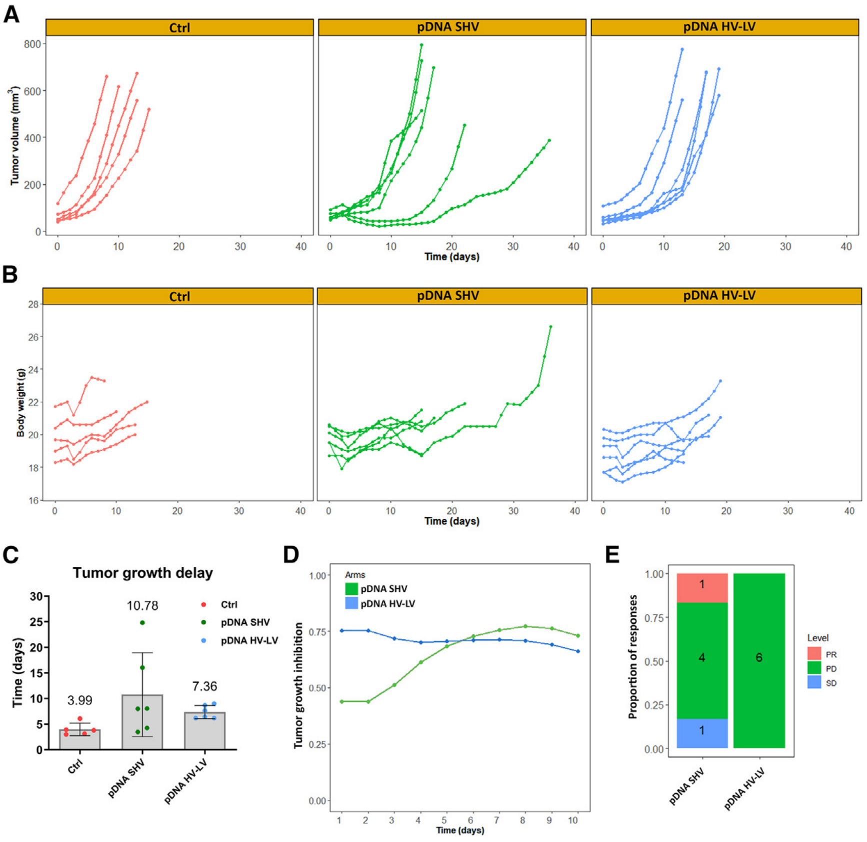

Figure 1. *In vivo* antitumor effect of pDNA GET using the SHV or HV-LV pulse protocol in CT26 tumors
(A) Individual growth curves of the tumor volume, (B) body weight of the mice, (C) tumor growth delay calculated via the tumor doubling time, (D) tumor growth inhibition and (E) responses after pDNA GET via the SHV or HV-LV pulse protocol. PD, progressive disease; PR, partial response; SD, steady disease. Data presented as the arithmetic mean (AM) ± standard deviation (SD) ($n \geq 5$). Statistical significance determined by one-way ANOVA.

may influence transfection efficiency in a panel of four cell lines in terms of activation of the innate immune response to *in vitro* transfection of pDNA.[23]

This study represents an important step forward in understanding the molecular mechanisms involved in the modulation of both the innate and adaptive immune responses and in the activation of various cell death pathways after the *in vivo* application of EP protocols for genetic transfer. We first performed RNA-seq analysis on tumors injected with noncoding pDNA and electroporated via two different EP protocols for GET approaches. As emphasized in previous studies,[22,24–26] the EP-mediated administration of control, noncoding pDNA in gene therapy studies provides a suitable platform for understanding the fundamental responses to GET-based approaches.

The first protocol tested in our study was based on short- and high-voltage (SHV) electric pulses, which have been successfully used for gene therapy with pDNA encoding human interleukin-12 (IL-12) in clinical trials, following preclinical studies that demonstrated its efficacy.[14,15,27–30]

The SHV pulse protocol was selected because it has been successfully used in clinical GET trials[14–18,27] and is already used in clinical settings in Europe for ECT[31,32]; therefore, it could be easily adapted for use in GET approaches in Europe. Its wide recognition also makes it a valuable reference for comparing other pulse protocols. Our second protocol, which is based on high-voltage-low-voltage (HV-LV) electric pulses, was adapted from Forjanic et al., where a similar combination of high and medium voltages resulted in high and long-term transfection efficiency of reporter genes in mouse skin.[33]

We studied the ability of these two electric pulse conditions to activate immune and tissue responses in murine CT26 colorectal tumors treated with intratumoral noncoding pDNA EP rather than an antigen or immunostimulatory molecule. Leukocyte infiltration in tumors was evaluated after both treatments, and RNA-seq analysis was performed to assess gene expression changes induced by GET, with a specific focus on immune and cellular signaling pathways.

RESULTS

Intratumoral plasmid GET using SHV and HV-LV electric pulse protocols results in similar effects on tumor growth

The effects of pDNA transfection via the SHV and HV-LV pulse protocols on tumor growth in a CT26 murine colon cancer model were evaluated *in vivo*. Drug-response analysis of the patient-derived xenograft (PDX) platform (DRAP) following GET revealed delayed tumor growth in both GET-treated groups compared with the control (Ctrl) group, but this difference did not reach statistical significance (Figure 1A). Tumor growth was similar among individual mice in both groups, except for two mice treated with pDNA via the SHV protocol, which exhibited a prolonged delay in tumor growth. None of the treatments affected the body weights of the mice (Figure 1B). The mean tumor volume doubling times (DTs) for the pDNA SHV and pDNA HV-LV groups were 10.78 (±8.19) and 7.36 (±1.28) days, respectively (Figure 1C). In addition, in the first 5 days after treatment, the calculated tumor growth inhibition (TGI) differed between the treatment groups, with a more prominent TGI observed in the pDNA HV-LV group (Figure 1D). However, owing to the prolonged tumor growth delay of the two groups of mice in the pDNA SHV group, the TGI increased in the days following and became greater than that in the pDNA HV-LV group. The treatment responses of

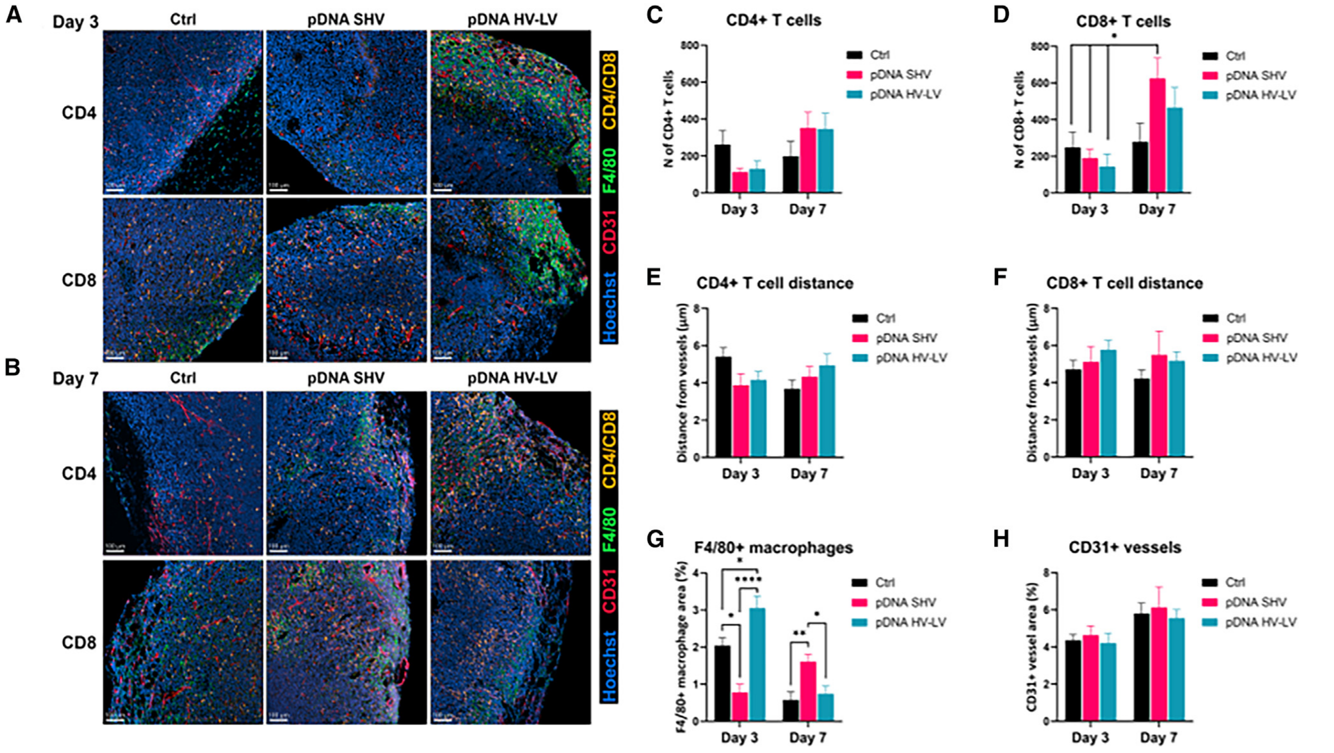

Figure 2. Immune cell infiltration at the tumor edges on days 3 and 7 after pDNA GET in CT26 murine tumors via the SHV or HV-LV pulse protocol

(A and B) Immunofluorescence analysis (cell nuclei, blue; anti-CD4 or anti-CD8, yellow; anti-CD31, red; anti-F4/80, green) on tumors collected on (A) day 3 and (B) day 7 after GET for histological analysis. Scale bar: 100 mm. (C–H) Quantification of immunofluorescence data presented as the AM ± SD ($n \geq 5$). Statistical significance was determined by one-way ANOVA. Adjusted $p < 0.05$ was considered to indicate statistical significance (*$p < 0.05$, **$p < 0.01$, ****$p < 0.0001$ vs. control, untreated cells or tumors [Ctrl]) between treatments or time points.

the mice were assessed based on Novartis Institutes for BioMedical Research PDX encyclopedia (NPDXE) response criteria from the DRAP package. In the pDNA SHV group, DRAP analysis identified and separated the two groups of mice with prolonged tumor growth delay—one with a partial response and the other with stable disease (Figure 1E). All other responses were identified as progressive disease. In the pDNA HV-LV group, all the responses were identified as progressive disease.

CT26 tumors show differential infiltration of cytotoxic T cells and macrophages after pDNA GET via the SHV or HV-LV pulse protocol

To determine the effect of pDNA GET on CT26 tumors via SHV or HV-LV pulse protocols on the recruitment of immune cell subpopulations, immunofluorescence analysis of $CD4^+$ helper and $CD8^+$ cytotoxic T cells, as well as $F4/80^+$ macrophages, was performed on tumor tissues collected 3 and 7 days after therapy (Figure 2). On day 3, image analysis of tumor edges revealed an initial decrease in the number of $CD4^+$ and $CD8^+$ T cells compared with that in control tumor tissues, regardless of the electric pulse protocol used (Figures 2A and 2B). However, on day 7, an increase in $CD4^+$ T cells and an even greater increase in $CD8^+$ T cells were observed in both treatment groups. In particular, the SHV pulse protocol led to a significant increase in $CD8^+$ T cells on day 7 compared with those in all the other groups on day 3 (Figures 2C and 2D; Table S3). To investigate whether there was a spatial correlation between infiltrated immune cells and tumor vessels, we also stained $CD31^+$ endothelial cells and measured the distance from $CD4^+$ and $CD8^+$ T cells to tumor vessels (Figures 2E and 2F). None of the tested electric pulse protocols affected the distance of $CD4^+$ and $CD8^+$ T cells to the tumor vessels at either time point.

Among the most notable changes observed after the treatment was also the number of $F4/80^+$ macrophages (Figure 2G). Interestingly, the SHV pulse protocol led to a significant decrease in macrophage infiltration on day 3 after therapy, which was restored on day 7 and significantly increased compared with that of the control. The opposite trend was observed for the HV-LV pulse protocol; on day 3, the area of macrophage infiltration was significantly greater than that of the control, but on day 7, it decreased and was similar to that of the control. Additionally, the HV-LV pulse protocol on day 3 resulted in a significant increase in macrophage recruitment compared with that in all the other groups on day 7 (Figure 2G; Table S4).

$CD31^+$ endothelial cells were not significantly affected by the treatment, although the area of the tumor vessels slightly increased on

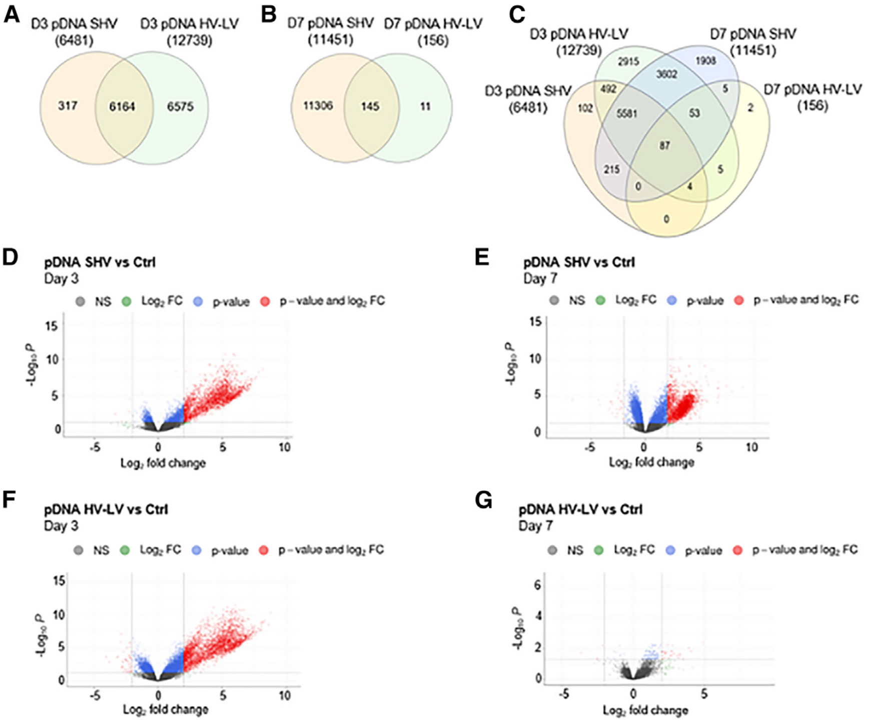

Figure 3. Differential gene expression analysis in CT26 murine tumors treated with pDNA GET via the SHV or HV-LV pulse protocol

(A–C) Venn diagrams showing the distribution of DEGs among pairwise comparisons. DEGs with adjusted $p < 0.05$ were considered significant. (D–G) Volcano plots showing significant gene expression changes between the control group and the groups of tumors that received pDNA GET via the SHV and HV-LV pulse protocols.

day 7 (Figure 2H). Compared with those at the tumor edge, there were no significant changes in the numbers of investigated immune cell populations in the tumor core after treatment (Figures S1 and S2).

Genome-wide expression analysis

Genome-wide expression analysis was performed to identify genes regulated by both pDNA GET treatments (i.e., the SHV and HV-LV electric pulse protocols). Transcriptome analysis on day 3 after treatment revealed 12,739 differentially expressed genes (DEGs) in tumors treated with the HV-LV pulse protocol and 6,481 DEGs in tumors treated with the SHV pulse protocol compared with those in the controls (Figure 3A). A total of 6,164 DEGs were shared by both treatments. Transcriptome analysis on day 7 revealed the opposite trend: compared with the control protocol, the SHV pulse protocol generated 11,451 DEGs, whereas the HV-LV pulse protocol generated only 156 DEGs in treated tumors (Figure 3B). A total of 145 DEGs were shared by both treatments. Considering both time points of analysis (i.e., day 3 and day 7 after applying the GET protocols), we also obtained the number of DEGs shared across all the experimental conditions (Figure 3C). For example, tumors treated with the SHV pulse protocol on day 3 and day 7 presented 5,883 DEGs, whereas tumors treated with the HV-LV pulse protocol on day 3 and day 7 presented 149 DEGs.

Volcano plots are shown to provide an overview of the differential expression of genes between tumors injected with pDNA and those exposed to SHV (Figures 3D and 3E) or HV-LV (Figures 3F and 3G) pulse protocols on day 3 and day s7even, respectively.

Functions related to Cell Death and Survival and Cell Signaling and Metabolism are modified in CT26 tumors after pDNA GET via the SHV electric pulse protocol

Several cellular and immune signaling pathways were differentially regulated after pDNA EP via SHV or HV-LV pulse protocols, and distinct signaling pathways switched between functions and/or overlapped into common networks.

We investigated the effective clustering of functional genes via enrichment analysis of Kyoto Encyclopedia of Genes and Genomes (KEGG) pathways.[34] In particular, the most upregulated KEGG pathways in tumors treated with the pDNA SHV pulse protocol 3 days after treatment were related to Immune Cell Trafficking (S100 Family Signaling Pathway), Cellular Growth and Proliferation (G-Protein-Coupled Receptor Signaling), and Cell Signaling and Molecular Transport (Cyclic AMP-Mediated Signaling and Calcium Signaling) (positive Z score and $-\log(p \text{ value}) > 1.6$) (Figure 4A). In contrast, the most downregulated KEGG pathways were related to Protein Signaling and Cell Death and Survival (EIF2 Signaling) (negative Z score and $-\log(p \text{ value}) = 3.1$), followed by BEX2 Signaling Pathway ($-\log(p \text{ value}) = 3$) and Developmental Disorder and Metabolic Disease (Oxidative Phosphorylation) ($-\log(p \text{ value}) = 1.7$) (Figure 4A).

For the same experimental group analyzed 7 days after the application of the SHV pulse protocol, the most enriched KEGG pathway in treated tumors was related to Cell-to-Cell Signaling and Interaction (Granzyme A Signaling) (positive Z score and $-\log(p \text{ value}) = 3.6$); conversely, the most downregulated KEGG pathway was associated with Protein Signaling, Cell Death and Survival (EIF2 Signaling) (negative Z score and $-\log(p \text{ value}) = 7.6$) (Figure 4B). In addition, significant differential activation of other signaling pathways related to metabolic reactions was observed in this group. In particular, significant downregulation of Serine and Glycine Biosynthesis, Purine Nucleotides *De Novo* Biosynthesis II, Phosphatidylglycerol Biosynthesis II, and Superpathway of Cholesterol Biosynthesis was observed (negative Z score and $-\log(p \text{ value}) > 1.3$) (Figure 4B).

Focusing on cell functions, we observed a shift from the initial activation of functions related to the Cellular Immune Response, Cancer, Intracellular, and Second Messenger Signals and Cellular Stress and

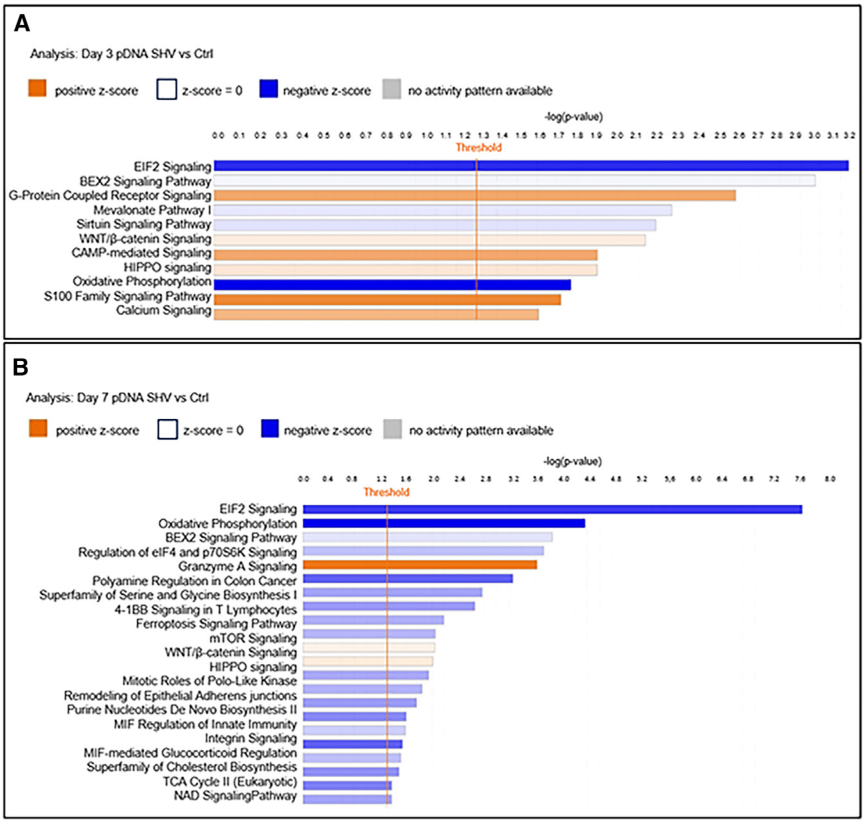

Figure 4. Enriched GO canonical pathways in the pDNA SHV group

Analysis of tumors treated with pDNA GET via the SHV pulse protocol compared with controls at (A) 3 days and (B) 7 days. The plot shows the GO molecular function terms plotted in order of significance.

Injury 3 days after pDNA GET via the SHV pulse protocol to the inactivation of these same functions by day 7 after treatment. At this time point, we found a downregulation of functions related to Intracellular and Second Messenger Signal, Cellular Stress and Injury, Cell Growth, Proliferation, and Development, along with moderate activation of functions related to Cellular Immune Response (Figures 5A and 5B).

Functions related to Cell Death and Survival, Cell Signaling, and Immune Response are modified in CT26 tumors after pDNA GET via the HV-LV electric pulse protocol

Similar to the findings in tumors subjected to pDNA GET via the SHV pulse protocol, tumors treated with the HV-LV pulse protocol presented significant downregulation of KEGG pathways related to Protein Signaling, Cell Death and Survival (EIF2 Signaling) 3 days after treatment (negative Z score and $-\log(p \text{ value}) = 16$). This was followed by various metabolic pathways (Superpathway of Cholesterol Biosynthesis, Mevalonate Pathway, Cholesterol Biosynthesis), Cell Cycle Control of Chromosomal Replication and Oxidative Phosphorylation ($-\log(p \text{ value}) > 1.5$) (Figure 6A). In contrast, the most upregulated KEGG pathway was related to Immune Cell Trafficking (S100 Family Signaling Pathway) (positive Z score and $-\log(p \text{ value}) > 2.5$), followed by pathways related to mitochondrial dysfunction (positive Z score and $-\log(p \text{ value}) > 2.5$) and Cell Signaling and Molecular Transport (Calcium Signaling and Granzyme A Signaling) (positive Z score and $-\log(p \text{ value}) > 1.5$) (Figure 6A).

In the same experimental group analyzed on day 7, the most upregulated KEGG pathway in treated tumors was the S100 Family Signaling Pathway, which reached a very high level of statistical significance ($-\log(p \text{ value}) > 19.5$). This was followed by several metabolic pathways also observed in the other experimental groups (Superpathway of Cholesterol Biosynthesis, Cholesterol Biosynthesis, Zymosterol Biosynthesis, and Glycolysis I) (negative Z score and $-\log(p \text{ value}) > 2.5$) (Figures 6B and S3B). Moreover, this group exhibited differential activation and inactivation of several pathways related to immune functions, such as the Th1 Pathway, IL-12 Signaling and Production in Macrophages, Th2 Pathway, the GP6 Signaling Pathway, Natural Killer Cell Signaling, and Pathogen Induced Cytokine Storm Signaling Pathway (positive Z score and $-\log(p \text{ value}) > 6$), as well as T Cell Receptor Signaling, TEC Kinase Signaling, ICOS-ICOSL Signaling in T Helper Cells, CD28 Signaling in T Helper Cells, S100 Family Signaling Pathway, IL-4 Signaling, Crosstalk Between Dendritic Cells and Natural Killer Cells, and Leukocyte Extravasation Signaling and Phagosome Formation (positive Z score and $-\log(p \text{ value}) > 3$) (Figures 6B and S3B). Interestingly, there was also a significant involvement of pathways related to Cellular Assembly and Organization, such as the upregulation of FAK Signaling, Actin Cytoskeleton Signaling, and Wound Healing Signaling Pathway (positive Z score and $-\log(p \text{ value}) > 3$) (Figures 6B and S3B).

With respect to cell functions, a similar trend was observed in the activation of functions previously observed in tumors treated with pDNA GET via the SHV pulse protocol at both 3 and 7 days after treatment. However, a significant difference was detected on day 7 after treatment. At this time point, there was strong activation of functions related to Cell Cycle Regulation, Intracellular and Second Messenger Signaling, Cellular Stress and Injury, Cellular Growth, and Proliferation and Development compared to the moderate activation of these functions in tumors treated with pDNA GET using SHV pulse protocol at the same time point. Moreover, there was a shift from the initial activation of functions related to Cellular Immune Response, Cancer, Intracellular and Second Messenger Signal, and Cellular Stress and Injury in tumors analyzed three days after pDNA GET via the SHV pulse protocol to the inactivation of these same functions 7 days after treatment. At this later time point, a downregulation of functions

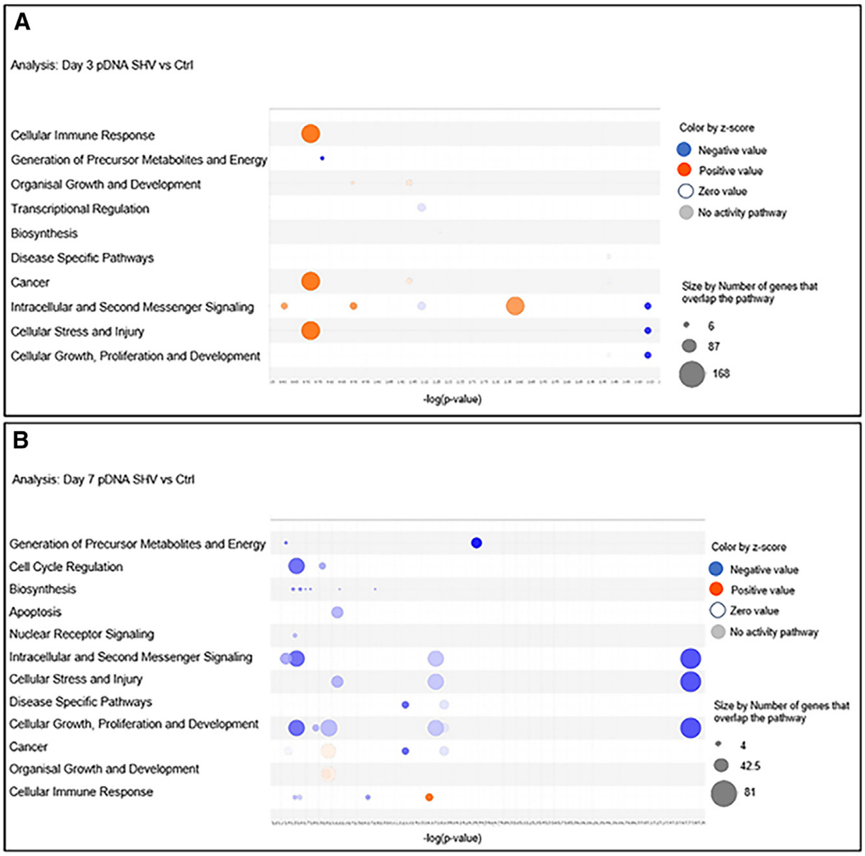

Figure 5. Bubble plot of the top cell functions per *Z* score in the pDNA SHV group

Analysis of tumors treated with pDNA GET via the SHV pulse protocol at (A) 3 days and (B) 7 days. A *Z* score ≥ |2| was used as the cutoff. Each bubble represents a function, and the bubble size is directly proportional to the −log (corrected *p* value)—in other words, the larger the bubble size, the more significant the result. The bubble color represents the *Z* score per the legend.

related to Intracellular and Second Messenger Signal, Cellular Stress and Injury, and Cell Growth, Proliferation, and Development was observed, with moderate activation of functions related to the Cellular Immune Response (Figures 7A and 7B).

DISCUSSION

The delivery of naked DNA to cells can activate various signaling pathways, leading to immune responses and different cell signaling pathways. When pDNA enters a cell, it can activate several pathways that sense the innate immune response, such as Toll-like receptor signaling, the cyclic guanosine monophosphate-adenosine monophosphate synthase-stimulator of interferon genes pathway, and the AIM2 inflammasome.[35,36]

Previous studies have investigated gene expression changes after intramuscular pDNA electrotransfer, testing the use of electric pulses in combination with or without pDNA injection.[21,37] These studies demonstrated that the application of electric pulses alone to muscle induces the expression of short-term chemokines as well as several stress-related mRNAs. However, the combination of pDNA injection and EP induces more significant chemokine expression[21] and an inflammatory response,[25] especially 7 days after intramuscular pDNA electrotransfer.[25] These studies also reported slightly divergent results depending on the different time points assayed, such as acute responses a few hours posttreatment and more chronic effects of pDNA EP at later time points.

To our knowledge, this is the first report describing the effects of two different EP protocols, in combination with noncoding pDNA directly injected into tumors by electrotransfer, in terms of immune cell recruitment and transcriptomic profiling.

We tested two noncoding pDNA EP protocols in mice based on SHV and HV-LV parameters to study how different electric pulse protocols applied directly to tumors can influence the outcome of EP-based treatments. Both protocols were evaluated on day 3 and day 7 posttreatment. These two time points were chosen to study the early innate immune response, which is typically established within the first few days, as well as the adaptive immune response, which usually occurs 5 days after treatment.[38]

The antitumor effect of pDNA GET via SHV and HV-LV pulse protocols on a CT26 murine colon cancer model was observed by measuring tumor growth after treatment. DRAP analysis revealed delayed tumor growth in both treatment groups compared with the control group, suggesting a possible role of both GET treatments alone (i.e., in the absence of an antigen or an immunomodulatory molecule) in affecting tumor development. In particular, TGI initially differed between the treatment groups, with the most prominent TGI in the pDNA HV-LV group. However, owing to the prolonged tumor growth delay of the two mice in the pDNA SHV group, the TGI in the days following increased and was greater than that in the pDNA HV-LV group. Moreover, DRAP analysis was able to distinguish and categorize different types of responses following treatment with the SHV pulse protocol, identifying partial response and stable disease response. In the pDNA HV-LV group, all treatment responses were identified as progressive disease. These results indicate that although the tested GET pulse protocols are similar, the SHV pulse protocol led to a better overall response than the HV-LV pulse protocol did, but the difference did not reach statistical significance. Therefore, we cannot highlight differences between the two GET protocols in counteracting tumor development in the absence of immunomodulatory molecules.

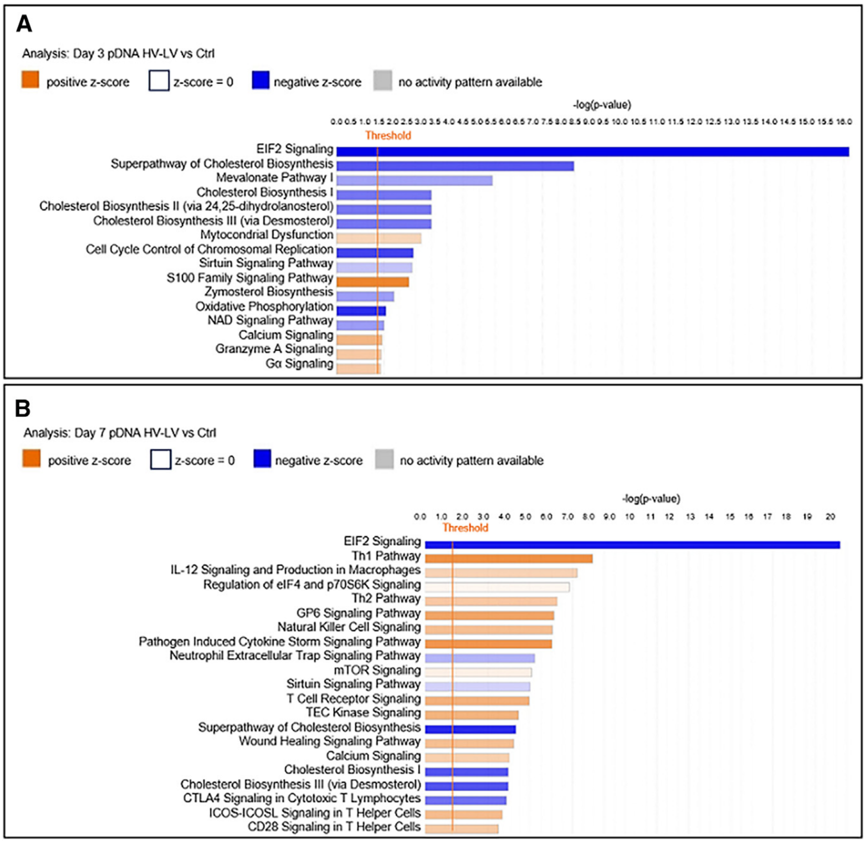

Figure 6. Enriched GO canonical pathways in the pDNA HV-LV group

Analysis of tumors treated with pDNA GET via the HV-LV pulse protocol compared with control tumors at (A) 3 days and (B) 7 days. The plot shows the GO molecular function terms plotted in order of significance.

Although the proportions of different immune cell subsets infiltrating CT26 tumors vary among studies, Mosely and colleagues defined the CT26 tumor model as immunoinflammatory, characterized by high infiltration of natural killer (NK) (>26%) and T cells (>18%), among which $CD8^+$ cytotoxic T lymphocytes (CTLs) are predominant (>5%).[39] The effect of noncoding pDNA delivery via GET pulses (8 square electric pulses with a voltage-to-distance ratio of 600 V/cm, a pulse duration of 5 ms, and a frequency of 1 Hz) on NK cell and CTL infiltration in B16F10 tumors was previously reported by Savarin et al.[40] Immunohistochemical staining on day 6 after therapy revealed an increased number of immune cells after 3× GET of noncoding pDNA compared with the untreated control. Interestingly, the application of 3× GET pulses alone (without pDNA) did not result in statistically significant differences. As a result, to a certain degree, a similar effect on the antitumor immune response was ascribed to the electrical pulses alone and to noncoding pDNA GET in this tumor model.

In this study, we evaluated the impact of pDNA GET on CT26 tumors via either SHV or HV-LV pulse protocols, with a focus on the recruitment of different immune cell populations. Immunofluorescence staining of helper $CD4^+$ and cytotoxic $CD8^+$ T cells in tumor tissues collected 3 and 7 days after treatment revealed a significant increase in $CD8^+$ T cells in tumors treated with the SHV pulse protocol on day 7 compared with those in all groups on day 3. This delayed increase in $CD8^+$ T cells might be attributed to the slow processing and presentation of antigens following tumor cell death.[41] Additionally, compared with the HV-LV protocol, the SHV protocol likely facilitates more consistent antigen and damage-associated molecular patterns (DAMPs) release and improved antigen presentation, resulting in stronger $CD8^+$ T cell responses.[42] Neither pulse protocol significantly affected the proximity of $CD4^+$ and $CD8^+$ T cells to $CD31^+$ endothelial cells in the tumor vessels, nor did they alter the area of the tumor vessels at any of the examined time points. These findings suggest that the primary factor influencing immune cell dynamics in our setting is the direct impact of pDNA GET on cellular viability and the release of immune attractants rather than changes in vascularization or endothelial activation.

Notably, the most evident changes were observed in macrophage recruitment following the SHV and HV-LV pulse protocols. Compared with the control protocol, the SHV pulse protocol caused a significant reduction in the number of macrophages on day 3 posttreatment, followed by a significant increase by day 7. In contrast, the HV-LV pulse protocol resulted in an initial significant increase in macrophage recruitment on day 3 compared with that of the control, which decreased by day 7 to levels like those of the control group. The early decrease and subsequent increase in macrophages in the SHV protocol may reflect the fluctuations observed in $CD8^+$ T cell numbers, likely due to the direct cytotoxic effects of the electrical pulses, followed by a recovery phase in which macrophages are recruited for debris clearance. However, the HV-LV protocol, which is less cytotoxic, leads to quicker but more transient macrophage infiltration for similar reasons.

In addition, we investigated the number of immune cells within the tumor center, which is known to be more difficult to penetrate than the tumor vessels compared to the tumor edge.[43,44] However, we observed no significant differences in the number of immune cells between the treated and control samples.

Taken together, these findings illustrate a dynamic immune landscape post-GET, where initial cellular damage and subsequent immune responses lead to varied temporal patterns of immune cell presence. Specifically, although specific differences due to the two treatments were observed, our investigation revealed a shift in the immune cell

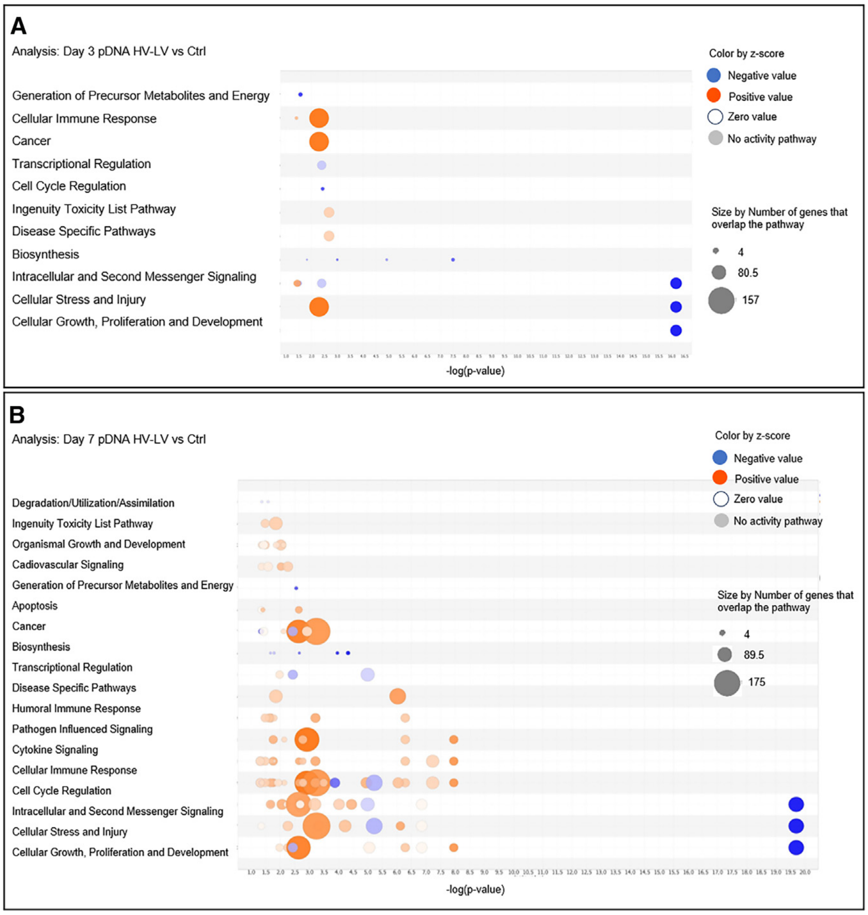

Figure 7. Bubble plot of the top cell functions per *Z* score in the pDNA HV-LV group

Analysis of bubble plots of the top cell functions per *Z* score in tumors treated with pDNA GET via the HV-LV pulse protocol at (A) 3 days and (B) 7 days. A *Z* score $\geq |2|$ was used as the cutoff. Each bubble represents a function, and the bubble size is directly proportional to the −log (corrected *p* value)—in other words, the larger the bubble size, the more significant the result. The bubble color represents the *Z* score per the legend.

equilibrium of CT26 tumors toward a more inflamed profile following GET, indicating a greater presence of immune cells than in untreated controls. These findings suggest a potential transition toward a more favorable immune microenvironment, enhanced by both the SH and HV-LV pulse protocols, with potential implications for therapeutic interventions targeting tumor immunity. However, we acknowledge that immunofluorescence analysis alone may not fully capture the complexity of the tumor microenvironment. Incorporating additional methods, such as flow cytometry or protein analysis, could provide more quantitative and objective data.

To further clarify the underlying molecular mechanisms driving the observed growth delay and immune responses, we analyzed the differences in the transcriptomic profiles of tumors treated with pDNA GET via either the SHV or HV-LV pulse protocol. The transcriptomic analysis revealed significant changes in gene expression, with important differences depending on the protocol and time point investigated. Among the most downregulated pathways in both SHV- and HV-LV-treated tumors there was EIF2 Signaling, which is crucial for the global initiation of mRNA translation in eukaryotic cells in response to various environmental stresses.[45] This finding aligns with previous evidence,[46] suggesting that the entry of heterologous pDNA into the cell, coupled with EP-induced stress, may inhibit protein synthesis by affecting mRNA translation via EIF2. This mechanism is physiologically linked to host cell responses to viral infection[47] and is critical when a GET strategy is planned to increase the expression of recombinant proteins.

The S100 Family Signaling Pathway was among the most upregulated pathways in tumors treated by pDNA GET using both SHV and HV-LV pulse protocols 3 days after the treatment. The primary function of S100 proteins is to act as a DAMP and activate immune functions,[48] thus orchestrating both innate and adaptive immune responses.[49] Therefore, activating DAMPs in the context of GET protocol application could be beneficial (see below).

We found, however, that most of the up- or downregulated signaling pathways in the SHV-treated tumors analyzed 3 and 7 days after treatment were related to Cell Signaling, Cell Death and Survival, and Metabolism. We recorded a time-dependent, differential activation of those pathways, with the former occurring early (3 days after GET) and the latter increasing at later time points.

Specifically, we observed downregulation of the BEX2 Signaling Pathway, which is critical for modulating apoptosis in various cancer cell types,[50–52] suggesting that in our experiments, the SHV pulses might have caused apoptosis via plasma membrane damage. Thus, we can assume that apoptosis is a predominant mode of cell death after the application of SHV pulses, causing increased permeability that is ultimately instrumental in facilitating intracellular drug accumulation.[53]

Furthermore, while apoptosis is traditionally considered an immunologically silent or even tolerogenic physiological cell death pathway,[54] recent studies have revealed crucial interactions between apoptotic tumor cells and some components of the immune system. These findings indicate that exogenous stimuli can induce immunogenic types of apoptosis.[55,56] Their findings support our findings, suggesting that SHV electrical pulses can create an immunogenic microenvironment mediated by the apoptotic death of tumor cells.

As evidence of the significant activation of immunologic signals after the application of the SHV protocol and a significant increase in $CD8^+$ T lymphocyte infiltration, we observed an increase in Granzyme A Signaling 7 days after treatment. CTL and NK cells are key immune effectors that eradicate infected cells and tumor cells.[57] To destroy these targets, CTL and NK cells predominantly use the granule exocytosis pathway, releasing perforin and granzymes from cytolytic granules into the immunological synapse formed with the target.[58]

With respect to the transcriptomic profile of the tumors in the pDNA HV-LV groups, our results revealed that similar to those in the SHV setting, several pathways related to Cell Death and Survival, Cell Signaling, and Immune Response were dynamically modulated over time. Specifically, at 3 days, we observed the upregulation of Mitochondrial Dysfunction that along with the downregulation of Oxidative Phosphorylation (resulting in mitochondrial ATP depletion) may suggest the activation of necrotic cell death pathways. This finding is in line with several studies conducted in both *in vitro* and *in vivo* settings.[42] Necrosis, as well as necroptosis, pyroptosis, and ferroptosis, are forms of immunogenic cell death (ICD) that can trigger an immune response.[59] The increased infiltration of macrophages into the tumors observed in our study supports these transcriptomic results.

The HV setting may induce an earlier induction of necrosis than the SHV treatment does, with the latter triggering early apoptosis rather than necrosis.

However, the inclusion of pDNA and SHV/HV-LV pulsed electric field controls could have provided a clearer understanding of the distinct contributions of pDNA injection and pulsed electric fields to the immune response. Both the introduction of foreign DNA and the application of electric fields are known to trigger immune responses and gene expression changes, independent of the GET technique itself. The omission of these controls was primarily due to the study's focus on assessing the overall effects of combining noncoding pDNA injection and pulsed electric fields, with variations only in the EP protocols under evaluation.

In conclusion, our study confirmed the recruitment of immune cells in pDNA GET with both EP protocols. Furthermore, this study revealed for the first time which immunogenic cells are recruited and which genes are perturbed after pDNA GET treatment of tumors with two different electric pulse protocols (i.e., the SHV and HV-LV electric pulse protocols).

This analysis could help identify key information useful for selecting the best electrical parameters for GET-based antitumor immunization and gene therapy approaches. Many factors are crucial for successful treatment outcomes, including tissue type, duration, local or systemic gene expression, and the induction of different types of ICD. These factors may be crucial for therapeutically enhancing the immunogenicity of tumor cells and disrupting tumor-associated immunotolerance.

Our findings suggest that apoptosis is a predominant mode of cell death resulting from SHV pulse application. Compared with the other protocol, the delivery of the empty plasmid with this pulse protocol significantly increased the number of cytotoxic T cells 7 days after treatment, likely due to the induction of apoptotic tumor cell death and subsequent stronger antigen presentation.

In contrast, the combination of HV and LV electric pulses is particularly able to regulate the activation of cell death signaling represented by immunogenic necrotic pathways, as well as both the innate and adaptive immune response, as indicated by early macrophage infiltration and later T lymphocyte infiltration.

Future studies with knockout mice or high-throughput biological assays will be essential to disentangle each individual cellular function and understand both their role in modulating the immune response and how they influence successful EP-based treatment protocols.

MATERIALS AND METHODS

Plasmids

The plasmid pUNO1-mcs (pDNA) was purchased from InvivoGen (San Diego, CA) and amplified in competent *Escherichia coli* (JM109; Thermo Fisher, Waltham, MA). The plasmid was then isolated via the EndoFree Plasmid Mega kit (Qiagen, Hilden, Germany) according to the manufacturer's instructions. The concentration of the isolated plasmid was measured with a Qubit DNA Broad Range kit (Thermo Fisher) via fluorometric quantification via a Qubit 4 Fluorometer (Thermo Fisher). Plasmid quality was assessed by the 260/280 nm ratio via an Epoch Microplate Spectrophotometer (BioTek, Winooski, VT) and agarose gel electrophoresis. For the experiments, the plasmid was diluted in 0.9% NaCl saline to a final concentration of 2 μg/μL.

Mice

Six- to eight-week-old female inbred BALB/c mice (BALB/cAnNCrl) were obtained from Charles River Laboratories (Wilmington, MA). The mice weighed between 18 and 20 g at the beginning of the experiments and were kept in a specific pathogen-free environment with a 12-h light-dark cycle at 20°C–24°C with 55% ± 10% relative humidity and food and water provided *ad libitum*. The experiments were approved by the Ministry of Agriculture, Forestry, and Food of the Republic of Slovenia (permission no. 34401-1/2015/43). The experimental procedures were performed in compliance with the guidelines for animal experiments of the European Union (EU) directive (2010/63/EU) and ARRIVE (Animal Research: Reporting of *In Vivo* Experiments) guidelines.

The mice were randomly divided into the following experimental groups: 5 mice treated with GET via the SHV pulse protocol (SHV group), 6 mice treated with GET via the HV-LV pulse protocol (HV-LV group), and 6 nontreated control group (Ctrl). One day prior to the experiment, the backs of the mice were shaved. Tumors were grown on the backs of the mice after subcutaneous inoculation of 3×10^5 CT26 cells in 100 μL 0.9% NaCl saline. The tumor volume was

measured via a Vernier caliper and then calculated via a formula for ellipsoids ($a \times b \times c \times \pi/6$, where a, b and c are orthogonal tumor diameters). During the treatment, the mice were anesthetized with 2% (v/v) isoflurane (Piramal Health Care UK Ltd., Morpeth, UK). The GET procedure was performed when the tumor volume reached 50 mm^3.

After the treatments, tumor growth was measured three times per week. When the tumor volume reached 500 mm^3, the mice were euthanized. Additionally, the body weights of the mice and their behavior were assessed via the mouse grimace scale as an indicator of the systemic toxicity of the therapy.[60]

Intratumoral pDNA EP

The treatment was carried out by intratumoral injection of 50 μg of the pDNA. After a 5-min delay, GET was performed on the tumors via the application of electric pulses. Two different EP protocols for GET were used (Table S1). The SHV pulse protocol was based on an SHV electric pulse protocol (8 square-wave electric pulses, amplitude-to-distance ratio of 1,300 V/cm, duration of 100 μs, frequency of 1 Hz).[15] The second was the HV and low-voltage (LV) pulse protocol (4 trains of 1 HV + 1 LV square-wave electric pulses, amplitude-to-distance ratio of the HV electric pulses: 1,300 V/cm and LV electric pulses: 150 V/cm, duration HV: 100 μs and LV: 20 ms, frequency 1 Hz) adapted from Forjanic et al.[33] The pulses were generated by an ELECTRO Cell B10 electric pulse generator (Leroy Biotech, Toulouse, France) and were delivered via 6-mm parallel stainless-steel plate electrodes. During GET, a conductive gel (Gel G006 ECO; FIAB, Firenze, Italy) was used at the contact of the electrodes and the skin overlying the tumors to ensure good conductivity.

Tumor collection

On days 3 and 7 after treatment, the tumors (transfected with pDNA and nontreated controls) were collected for gene expression analysis and immunofluorescence staining. The mice were euthanized, and the tumors were surgically removed. Immediately after collection, half of the collected tumors were weighed and frozen in liquid nitrogen. Frozen tumor samples were crushed via a pestle and then stored at −80°C before RNA extraction. The other half of each tumor was first fixed in 4% paraformaldehyde (Thermo Fisher) overnight, then incubated in 30% sucrose for 24 h, embedded in optimal cutting temperature compound, and snap-frozen in liquid nitrogen.

RNA extraction

RNA was then extracted via the Total RNA Kit peqGOLD (VWR, Radnor, PA) according to the manufacturer's instructions. One microgram of total RNA sample was used for RNA quantification via a Qubit (Thermo Fisher), and quality checks were performed via TapeStation (Agilent Technologies, Santa Clara, CA).

RNA-seq

RNA-seq libraries were prepared from 700 ng of total RNA via the Illumina TruSeq Stranded Total RNA Sample Preparation Kit (Illumina, San Diego, CA) according to the manufacturer's protocol. The cDNA libraries were checked on a Bioanalyzer 2100 and quantified with a Qubit instrument (Thermo Fisher). RNA-seq was performed via the NovaSeq 6000 system (Illumina), which generated almost 100 million 150-bp paired-end reads per sample. In the preprocessing step, the raw reads in fastq format were inspected and cleaned via FASTP.[61] The mean quality per base was fixed at a phred score of 20 and reads with more than 30% ambiguous bases (-q 20 -u 30 -L 55 –detect_adapter_for_pe) were removed. Reads shorter than 55 bases were also removed. Cleaned reads were aligned with STAR (version 2.7.9a)[62] via the ENCODE standard options onto the primary version of the reference mouse genome (GRCm39). To correct for bias due to the heterogeneous patterns of transcript degradation in the RNA-seq data, R and the DegNorm pipeline were used.[63] Briefly, DegNorm processes the RNA-seq alignment files to calculate the coverage according to the gencode vM32 annotation, performs rank-one overapproximation on coverage matrices for each gene to estimate the degradation index (DI) score for each gene within each sample and outputs DI scores together with degradation-normalized read counts (based on DI scores). Normalization and differential gene expression analysis were conducted with DESeq2 (version 1.40.2)[64] via the contrast method. The volcano plots were generated with R via the EnhancedVolcano package. Venn diagrams were generated via the web-based tool InteractiVenn.[65]

Functional enrichment analysis

Gene Ontology (GO) enrichment analyses of the DEGs were performed via Ingenuity Pathway Analysis (IPA; Qiagen). This analysis was based on the calculation of *Z* scores, from which we inferred the activation states of the biological functions. This was possible when adjusted *p* values and log2(fold change) values accompanied the DEG lists submitted to IPA. An enrichment score (Fisher's exact test, "overlap *p* value") was calculated to measure the likelihood that an association between a set of DEGs and a related function was not due to chance. We considered $p < 0.05$ and *Z* scores >0 (activation) or <0 (inhibition).

Immunofluorescence

We prepared 14-μm-thick frozen tumor tissue sections via a CM1850 cryostat (Leica, Wetzlar, Germany) and stained with primary and secondary antibodies (Table S2). The sections were first dried for 10 min at 37°C and then washed twice for 5 min in 1× PBS. Antigen retrieval was then performed by placing the slides in hot citrate buffer (approximately 95°C), which was air cooled at room temperature (RT) for 30 min, followed by 30 min of cooling in RT water. After washing in 1× PBS, the sections were blocked/permeabilized in blocking buffer (0.5% Tween 20, 5% donkey serum, 22.52 mg/mL glycine in PBS) for 30 min at RT in a humidified chamber. Then, the sections were blocked for 1 h at RT in blocking buffer (5% donkey serum, 22.52 mg/mL glycine in PBS) and subsequently incubated with primary antibodies overnight in blocking buffer (2% donkey serum, 22.52 mg/mL glycine in 1× PBS) in a humidified chamber at 4°C. After being washed three times in 1× PBS, the sections were incubated with secondary blocking buffer (2% donkey serum, 22.52 mg/mL glycine in 1× PBS) for 1 h at RT in a humidified chamber and then washed three

times in 1× PBS. Nuclei were counterstained with Hoechst 33342 solution (3 μg/mL) (Thermo Fisher) in 1× PBS for 10 min in the dark. After another two washes in 1× PBS, the slides were mounted with ProLong Glass Antifade Mountant (Thermo Fisher). Three tumor samples per group were imaged with an LSM 800 confocal microscope (Carl Zeiss, Oberkochen, Germany) with a 20× objective (numerical aperture 0.8). Hoechst 33342, Alexa Fluor 488, Cy3, and Alexa Fluor 647 were excited with lasers with excitation wavelengths of 405, 488, 561, and 640 nm, respectively. To capture the emitted light, a gallium arsenide phosphide detector was used combined with variable dichroic filters at channel-specific wavelengths: 410–545 nm (Hoechst 33342), 488–545 nm (Alexa Fluor 488), 565–620 nm (Cy3), and 645–700 nm (Alexa Fluor 647). To ensure a representative analysis, we acquired three random images of frozen tumor tissue sections per sample—specifically two images of the tumor margin and one of the tumor centers. The images obtained were visualized and analyzed with Imaris software (Bitplane, Belfast, UK). The cutoff values for each channel were determined based on negative controls.

Statistical analysis

For statistical analysis and graph figures, R software (version 4.6.3, R Core Team, Vienna, Austria) and GraphPad Prism 10 (GraphPad Software, La Jolla, CA) were used. Tumor growth curves, mouse body weight curves, TGIs, and treatment responses were analyzed with the DRAP package[66] in R, while tumor growth delay, which was based on the tumor DT, was analyzed with GraphPad Prism 10. The DRAP package is defined as drug-response analysis on a PDX platform for four typical PDX trial designs. The package incorporates tools for data visualization, data analysis, and the presentation of findings. Specifically, the data analysis module enables us to statistically assess the difference in tumor volume between arms, calculate the TGI rate, and label the treatment response at the animal level.[66] Treatment responses of individual mice were classified based on the criteria of the NPDXE response criteria within the DRAP package.[67] The NPDXE response is determined by comparing the tumor volume change at time t to its baseline: % tumor volume change = ΔVolt = 100% × ((Vt – Vinitial)/Vinitial). The BestResponse is the minimum value of ΔVolt for t ≥ 10 d. For each time t, the average of ΔVolt from t = 0 to t is also calculated. The BestAvgResponse is defined as the minimum value of this average for t ≥ 10 d. This metric captures a combination of speed, strength, and durability of response into a single value. The criteria for response are defined as follows (applied in this order): complete response BestResponse < −95% and BestAvgResponse < −40%; partial response, BestResponse < −50% and BestAvgResponse < −20%; steady disease, BestResponse <35% and BestAvgResponse <30%; and progressive disease, not otherwise categorized (30). The TGI was calculated via the DRAP package as follows:

$$\mathrm{TGI} = \left(1 - \frac{F(\mathrm{V_T})}{F(\mathrm{V_C})}\right) * 100\%, \quad \text{(Equation 1)}$$

where $F(V_T)$ and $F(V_C)$ represent the area under the curve of the tumor volume of the treated and control groups.[68]

The statistical significance of the immunofluorescence data of the frozen tumor tissue sections was determined via one-way analysis of variance (ANOVA) with Dunnett's multiple comparisons post hoc test. The RNA-seq data were analyzed with R via one-way, two-way, and mixed-design ANOVA, the linear mixed model, the Wald test, and the t test. Non-normally distributed data were analyzed with nonparametric tests (Wilcoxon test, Mann-Whitney test, and Kruskal-Wallis test). A $p < 0.05$ was considered to indicate statistical significance ($^{*}p < 0.05$, $^{**}p < 0.01$, $^{***}p < 0.001$, $^{****}p < 0.0001$ vs. control, untreated cells, or tumors [Ctrl]).

DATA AND CODE AVAILABILITY

The data that support the findings of this study are available from the corresponding authors upon reasonable request. The raw RNA-seq data obtained and discussed in this publication have been deposited in the NCBI Sequence Read Archive (SRA) under the accession number SRP497902.

ACKNOWLEDGMENTS

We would like to thank Teja Valant (Institute of Oncology Ljubljana, Ljubljana, Slovenia) for her technical help. We also thank Dr. Matilde Paggiolu, technologist at IFT–CNR, for her support in this work; Dr. Ermes Filomena, technologist at the University of Bari, for his support in sharing the raw RNA-seq data on the NCBI-SRA repository; and Dr. Sharon Natasha Cox, researcher at the University of Bari, native English speaker, for her contribution to the English revision and editing of the manuscript. This project leverages the omics and computational facilities provided by the Italian Node of ELIXIR (ELIXIR-IT), the European Research Infrastructure for Life Science, including the advanced equipment acquired by the CNRBiOmics (PIR01_00017) and ELIXIRxNextGenIT (IR000010) infrastructural projects. This research was supported by: Slovenian Research Agency (ARIS), Slovenia, grant P3-0003; Project CNR - DSB.AD007.257, and Omics platform of ELIXIR-IT, Italy. Additionally, this work was undertaken within the ZAP Cancer project, which has received funding from the European Union's Horizon Europe Research and Innovation Programme under grant agreement no. 101160061.

AUTHOR CONTRIBUTIONS

M.D.R., E.S., and M.C. conceptualized, designed, and supervised the study; T.B. and B.M. optimized the methodology; M.D.R., T.B., and F.M. conducted the experiments; M.D.R., T.B., A.S., and I.S. analyzed and interpreted the data; M.D.R. and T.B. prepared the original manuscript draft; E.S., M.C., B.M., G.P., and A.T. reviewed and edited the manuscript; and M.C., E.S., and G.P. acquired the funding. All the authors have read and approved the final manuscript.

DECLARATION OF INTERESTS

The authors declare no competing interests.

SUPPLEMENTAL INFORMATION

Supplemental information can be found online at https://doi.org/10.1016/j.omtn.2025.102448.

REFERENCES

1. Cervia, L.D., and Yuan, F. (2018). Current Progress in Electrotransfection as a Nonviral Method for Gene Delivery. Mol. Pharm. *15*, 3617–3624.

2. Sokołowska, E., and Błachnio-Zabielska, A.U. (2019). Acritical review of electroporation as a plasmid delivery system in mouse skeletal muscle. Int. J. Mol. Sci. *20*, 12–15.

3. Luz, J.C.D.S.d., Antunes, F., Clavijo-Salomon, M.A., Signori, E., Tessarollo, N.G., and Strauss, B.E. (2021). Clinical applications and immunological aspects of electroporation-based therapies. Vaccines *9*, 727–822.

4. Sayed, N., Allawadhi, P., Khurana, A., Singh, V., Navik, U., Pasumarthi, S.K., Khurana, I., Banothu, A.K., Weiskirchen, R., and Bharani, K.K. (2022). Gene therapy: Comprehensive overview and therapeutic applications. Life Sci. *294*, 120375.

5. Gary, E.N., and Weiner, D.B. (2020). DNA vaccines: prime time is now. Curr. Opin. Immunol. *65*, 21–27.

6. Heller, R., and Heller, L.C. (2015). Gene electrotransfer clinical trials. Adv. Genet. *89*, 235–262.

7. Tellado, M., De Robertis, M., Montagna, D., Giovannini, D., Salgado, S., Michinski, S., Signori, E., and Maglietti, F. (2023). Electrochemotherapy Plus IL-2+IL-12 Gene Electrotransfer in Spontaneous Inoperable Stage III–IV Canine Oral Malignant Melanoma. Vaccines *11*, 1033–1116.

8. Jacobs, L., De Smidt, E., Geukens, N., Declerck, P., and Hollevoet, K. (2020). DNA-Based Delivery of Checkpoint Inhibitors in Muscle and Tumor Enables Long-Term Responses with Distinct Exposure. Mol. Ther. *28*, 1068–1077.

9. Maglietti, F., Tellado, M., De Robertis, M., Michinski, S., Fernández, J., Signori, E., and Marshall, G. (2020). Electroporation as the immunotherapy strategy for cancer in veterinary medicine: State of the art in Latin America. Vaccines *8*, 537–619.

10. Ursic, K., Kos, S., Kamensek, U., Cemazar, M., Miceska, S., Markelc, B., Bucek, S., Staresinic, B., Kloboves Prevodnik, V., Heller, R., and Sersa, G. (2021). Potentiation of electrochemotherapy effectiveness by immunostimulation with IL-12 gene electrotransfer in mice is dependent on tumor immune status. J. Contr. Release *332*, 623–635.

11. Low, L., Mander, A., McCann, K., Dearnaley, D., Tjelle, T., Mathiesen, I., Stevenson, F., and Ottensmeier, C.H. (2009). DNA vaccination with electroporation induces increased antibody responses in patients with prostate cancer. Hum. Gene Ther. *20*, 1269–1278.

12. Vonderheide, R.H., Kraynyak, K.A., Shields, A.F., McRee, A.J., Johnson, J.M., Sun, W., Chintakuntlawar, A.V., Pawlicki, J., Sylvester, A.J., McMullan, T., et al. (2021). Phase 1 study of safety, tolerability and immunogenicity of the human telomerase (hTERT)-encoded DNA plasmids INO-1400 and INO-1401 with or without IL-12 DNA plasmid INO-9012 in adult patients with solid tumors. J. Immunother. Cancer *9*, e003019.

13. Aurisicchio, L., Fridman, A., Mauro, D., Sheloditna, R., Chiappori, A., Bagchi, A., and Ciliberto, G. (2020). Safety, tolerability and immunogenicity of V934/V935 hTERT vaccination in cancer patients with selected solid tumors: a phase I study. J. Transl. Med. *18*, 39–48.

14. Greaney, S.K., Algazi, A.P., Tsai, K.K., Takamura, K.T., Chen, L., Twitty, C.G., Zhang, L., Paciorek, A., Pierce, R.H., Le, M.H., et al. (2020). Intratumoral Plasmid IL12 Electroporation Therapy in Patients with Advanced Melanoma Induces Systemic and Intratumoral T-cell Responses. Cancer Immunol. Res. *8*, 246–254.

15. Algazi, A.P., Twitty, C.G., Tsai, K.K., Le, M., Pierce, R., Browning, E., Hermiz, R., Canton, D.A., Bannavong, D., Oglesby, A., et al. (2020). Phase II Trial of IL-12 Plasmid Transfection and PD-1 Blockade in Immunologically Quiescent Melanoma. Clin. Cancer Res. *26*, 2827–2837.

16. Algazi, A., Bhatia, S., Agarwala, S., Molina, M., Lewis, K., Faries, M., Fong, L., Levine, L.P., Franco, M., Oglesby, A., et al. (2020). Intratumoral delivery of tavokinogene telseplasmid yields systemic immune responses in metastatic melanoma patients. Ann. Oncol. *31*, 532–540.

17. Telli, M.L., Nagata, H., Wapnir, I., Acharya, C.R., Zablotsky, K., Fox, B.A., Bifulco, C.B., Jensen, S.M., Ballesteros-Merino, C., Le, M.H., et al. (2021). Intratumoral Plasmid IL12 Expands CD8+ T Cells and Induces a CXCR3 Gene Signature in Triple-negative Breast Tumors that Sensitizes Patients to Anti-PD-1 Therapy. Clin. Cancer Res. *27*, 2481–2493.

18. Bhatia, S., Longino, N.V., Miller, N.J., Kulikauskas, R., Iyer, J.G., Ibrani, D., Blom, A., Byrd, D.R., Parvathaneni, U., Twitty, C.G., et al. (2020). Intratumoral Delivery of Plasmid IL12 Via Electroporation Leads to Regression of Injected and Noninjected Tumors in Merkel Cell Carcinoma. Clin. Cancer Res. *26*, 598–607.

19. Sieni, E., Dettin, M., De Robertis, M., Bazzolo, B., Conconi, M.T., Zamuner, A., Marino, R., Keller, F., Campana, L.G., and Signori, E. (2020). The efficiency of gene electrotransfer in breast-cancer cell lines cultured on a novel collagen-free 3D scaffold. Cancers *12*, 1043.

20. De Robertis, M., Pasquet, L., Loiacono, L., Bellard, E., Messina, L., Vaccaro, S., Di Pasquale, R., Fazio, V.M., Rols, M.P., Teissie, J., et al. (2018). In vivo evaluation of a new recombinant hyaluronidase to improve gene electro-transfer protocols for dna-based drug delivery against cancer. Cancers *10*, 405.

21. Sales Conniff, A., Tur, J., Kohena, K., Zhang, M., Gibbons, J., and Heller, L.C. (2022). Transcriptomic Analysis of the Acute Skeletal Muscle Effects after Intramuscular DNA Electroporation Reveals Inflammatory Signaling. Vaccines *10*, 2037–2110.

22. Bhandary, M., Sales Conniff, A., Miranda, K., and Heller, L.C. (2022). Acute Effects of Intratumor DNA Electrotransfer. Pharmaceutics *14*, 2097.

23. Warga, E., Anderson, J., Tucker, M., Harris, E., and Elmer, J. (2023). Transcriptomic analysis of the innate immune response to in vitro transfection of plasmid DNA. Mol. Ther. Nucleic Acids *31*, 43–56.

24. Heller, L.C., and Coppola, D. (2002). Electrically mediated delivery of vector plasmid DNA elicits an antitumor effect. Gene Ther. *9*, 1321–1325.

25. Mann, C.J., Anguela, X.M., Montané, J., Obach, M., Roca, C., Ruzo, A., Otaegui, P., Mir, L.M., and Bosch, F. (2012). Molecular signature of the immune and tissue response to non-coding plasmid DNA in skeletal muscle after electrotransfer. Gene Ther. *19*, 1177–1186.

26. Simna, S.P., and Han, Z. (2022). Prospects of Non-Coding Elements in Genomic DNA Based Gene Therapy. Curr. Gene Ther. *22*, 89–103.

27. Daud, A.I., DeConti, R.C., Andrews, S., Urbas, P., Riker, A.I., Sondak, V.K., Munster, P.N., Sullivan, D.M., Ugen, K.E., Messina, J.L., and Heller, R. (2008). Phase I trial of interleukin-12 plasmid electroporation in patients with metastatic melanoma. J. Clin. Oncol. *26*, 5896–5903.

28. Heller, L., Merkler, K., Westover, J., Cruz, Y., Coppola, D., Benson, K., Daud, A., and Heller, R. (2006). Evaluation of toxicity following electrically mediated interleukin-12 gene delivery in a B16 mouse melanoma model. Clin. Cancer Res. *12*, 3177–3183.

29. Lucas, M.L., Heller, L., Coppola, D., and Heller, R. (2002). IL-12 plasmid delivery by in vivo electroporation for the successful treatment of established subcutaneous B16.F10 melanoma. Mol. Ther. *5*, 668–675.

30. Lucas, M.L., and Heller, R. (2003). IL-12 gene therapy using an electrically mediated nonviral approach reduces metastatic growth of melanoma. DNA Cell Biol. *22*, 755–763.

31. Mir, L.M., Gehl, J., Sersa, G., Collins, C.G., Garbay, J.R., Billard, V., Geertsen, P.F., Rudolf, Z., O'Sullivan, G.C., and Marty, M. (2006). Standard operating procedures of the electrochemotherapy: Instructions for the use of bleomycin or cisplatin administered either systemically or locally and electric pulses delivered by the CliniporatorTM by means of invasive or non-invasive electrodes. Eur J Cancer Suppl. *4*, 14–25.

32. Gehl, J., Sersa, G., Matthiessen, L.W., Muir, T., Soden, D., Occhini, A., Quaglino, P., Curatolo, P., Campana, L.G., Kunte, C., et al. (2018). Updated Standard Operating Procedures for Electrochemotherapy of Cutaneous Tumours and Skin Metastases. Acta Oncol. *57*, 874–882.

33. Forjanic, T., Markelc, B., Marcan, M., Bellard, E., Couillaud, F., Golzio, M., and Miklavci, D. (2019). Electroporation-Induced Stress Response and Its Effect on Gene Electrotransfer Efficacy: In Vivo Imaging and Numerical Modeling. IEEE Trans. Biomed. Eng. *66*, 2671–2683.

34. Briard, B., Place, D.E., and Kanneganti, T.D. (2020). DNA sensing in the innate immune response. Physiology *35*, 112–124.

35. Znidar, K., Bosnjak, M., Cemazar, M., and Heller, L.C. (2016). Cytosolic DNA Sensor Upregulation Accompanies DNA Electrotransfer in B16.F10 Melanoma Cells. Mol. Ther. Nucleic Acids *5*, e322.

36. Peng, B., Zhao, Y., Xu, L., and Xu, Y. (2007). Electric pulses applied prior to intramuscular DNA vaccination greatly improve the vaccine immunogenicity. Vaccine *25*, 2064–2073.

37. Hojman, P., Zibert, J.R., Gissel, H., Eriksen, J., and Gehl, J. (2007). Gene expression profiles in skeletal muscle after gene electrotransfer. BMC Mol. Biol. *8*, 56.

38. Taylor, M.A., Hughes, A.M., Walton, J., Coenen-Stass, A.M.L., Magiera, L., Mooney, L., Bell, S., Staniszewska, A.D., Sandin, L.C., Barry, S.T., et al. (2019). Longitudinal immune characterization of syngeneic tumor models to enable model selection for immune oncology drug discovery. J. Immunother. Cancer *7*, 328–416.

39. Mosely, S.I.S., Prime, J.E., Sainson, R.C.A., Koopmann, J.O., Wang, D.Y.Q., Greenawalt, D.M., Ahdesmaki, M.J., Leyland, R., Mullins, S., Pacelli, L., et al. (2017). Rational selection of syngeneic preclinical tumor models for immunotherapeutic drug discovery. Cancer Immunol. Res. *5*, 29–41.

40. Savarin, M., Kamensek, U., Cemazar, M., Heller, R., and Sersa, G. (2017). Electrotransfer of plasmid DNA radiosensitizes B16F10 tumors through activation of immune response. Radiol. Oncol. *51*, 30–39.

41. Galluzzi, L., Kepp, O., Hett, E., Kroemer, G., and Marincola, F.M. (2023). Immunogenic cell death in cancer: concept and therapeutic implications. J. Transl. Med. *21*, 162.

42. Batista Napotnik, T., Polajžer, T., and Miklavčič, D. (2021). Cell death due to electroporation – A review. Bioelectrochemistry *141*, 107871.

43. Huang, Y., Kim, B.Y.S., Chan, C.K., Hahn, S.M., Weissman, I.L., and Jiang, W. (2018). Improving immune-vascular crosstalk for cancer immunotherapy. Nat. Rev. Immunol. *18*, 195–203.

44. Melssen, M.M., Sheybani, N.D., Leick, K.M., and Slingluff, C.L., Jr. (2023). Barriers to immune cell infiltration in tumors. J. Immunother. Cancer *11*, e006401.

45. Boye, E., and Grallert, B. (2020). eIF2α phosphorylation and the regulation of translation. Curr. Genet. *66*, 293–297.

46. Underhill, M.F., Coley, C., Birch, J.R., Findlay, A., Kallmeier, R., Proud, C.G., and James, D.C. (2003). Engineering mRNA translation initiation to enhance transient gene expression in Chinese hamster ovary cells. Biotechnol. Prog. *19*, 121–129.

47. Hershey, J.W.B., Sonenberg, N., and Mathews, M.B. (2019). Principles of translational control. Cold Spring Harbor Perspect. Biol. *11*, a032607.

48. Kültz, D. (2005). Molecular and evolutionary basis of the cellular stress response. Annu. Rev. Physiol. *67*, 225–257.

49. Bertheloot, D., and Latz, E. (2017). HMGB1, IL-1α, IL-33 and S100 proteins: Dual-function alarmins. Cell. Mol. Immunol. *14*, 43–64.

50. Zhou, X., Meng, Q., Xu, X., Zhi, T., Shi, Q., Wang, Y., and Yu, R. (2012). Bex2 regulates cell proliferation and apoptosis in malignant glioma cells via the c-Jun NH2-terminal kinase pathway. Biochem. Biophys. Res. Commun. *427*, 574–580.

51. Ding, K., Su, Y., Pang, L., Lu, Q., Wang, Z., Zhang, S., Zheng, S., Mao, J., and Zhu, Y. (2009). Inhibition of apoptosis by downregulation of hBex1, a novel mechanism, contributes to the chemoresistance of Bcr/Abl+ leukemic cells. Carcinogenesis *30*, 35–42.

52. Naderi, A. (2019). Molecular functions of brain expressed X-linked 2 (BEX2) in malignancies. Exp. Cell Res. *376*, 221–226.

53. Mitchell, P. (1979). Keilin's respiratory chain concept and its chemiosmotic consequences. Science *206*, 1148–1159.

54. Green, D.R., Ferguson, T., Zitvogel, L., and Kroemer, G. (2009). Immunogenic and tolerogenic cell death. Nat. Rev. Immunol. *9*, 353–363.

55. Montico, B., Nigro, A., Casolaro, V., and Dal Col, J. (2018). Immunogenic Apoptosis as a Novel Tool for Anticancer Vaccine Development. Int. J. Mol. Sci. *19*, 594.

56. Obeid, M., Tesniere, A., Ghiringhelli, F., Fimia, G.M., Apetoh, L., Perfettini, J.L., Castedo, M., Mignot, G., Panaretakis, T., Casares, N., et al. (2007). Calreticulin exposure dictates the immunogenicity of cancer cell death. Nat. Med. *13*, 54–61.

57. Le, D.T., Huynh, T.R., Burt, B., van Buren, G., Abeynaike, S.A., Zalfa, C., Nikzad, R., Kheradmand, F., Tyner, J.J., and Paust, S. (2021). Natural killer cells and cytotoxic T lymphocytes are required to clear solid tumor in a patient-derived xenograft. JCI Insight *6*, 1–18.

58. Pardo, J., Balkow, S., Anel, A., and Simon, M.M. (2002). Granzymes are essential for natural killer cell-mediated and perf-facilitated tumor control. Eur. J. Immunol. *32*, 2881–2887.

59. Gamrekelashvili, J., Greten, T.F., and Korangy, F. (2015). Immunogenicity of necrotic cell death. Cell. Mol. Life Sci. *72*, 273–283.

60. Langford, D.J., Bailey, A.L., Chanda, M.L., Clarke, S.E., Drummond, T.E., Echols, S., Glick, S., Ingrao, J., Klassen-Ross, T., Lacroix-Fralish, M.L., et al. (2010). Coding of facial expressions of pain in the laboratory mouse. Nat. Methods *7*, 447–449.

61. Chen, S., Zhou, Y., Chen, Y., and Gu, J. (2018). Fastp: An ultra-fast all-in-one FASTQ preprocessor. Bioinformatics *34*, i884–i890.

62. Dobin, A., Davis, C.A., Schlesinger, F., Drenkow, J., Zaleski, C., Jha, S., Batut, P., Chaisson, M., and Gingeras, T.R. (2013). STAR: Ultrafast universal RNA-seq aligner. Bioinformatics *29*, 15–21.

63. Xiong, B., Yang, Y., Fineis, F.R., and Wang, J.P. (2019). DegNorm: Normalization of generalized transcript degradation improves accuracy in RNA-seq analysis. Genome Biol. *20*, 75.

64. Love, M.I., Huber, W., and Anders, S. (2014). Moderated estimation of fold change and dispersion for RNA-seq data with DESeq2. Genome Biol. *15*, 550–621.

65. Heberle, H., Meirelles, G.V., da Silva, F.R., Telles, G.P., and Minghim, R. (2015). InteractiVenn: A web-based tool for the analysis of sets through Venn diagrams. BMC Bioinf. *16*, 169–177.

66. Li, Q., Dai, W., Liu, J., Li, Y.X., and Li, Y.Y. (2019). DRAP: A toolbox for drug response analysis and visualization tailored for preclinical drug testing on patient-derived xenograft models. J. Transl. Med. *17*, 39.

67. Gao, H., Korn, J.M., Ferretti, S., Monahan, J.E., Wang, Y., Singh, M., Zhang, C., Schnell, C., Yang, G., Zhang, Y., et al. (2015). High-throughput screening using patient-derived tumor xenografts to predict clinical trial drug response. Nat. Med. *21*, 1318–1325.

68. Hoeflich, K.P., Merchant, M., Orr, C., Chan, J., Den Otter, D., Berry, L., Kasman, I., Koeppen, H., Rice, K., Yang, N.Y., et al. (2012). Intermittent administration of MEK inhibitor GDC-0973 plus pi3k inhibitor GDC-0941 triggers robust apoptosis and tumor growth inhibition. Cancer Res. *72*, 210–219.

OMTN, Volume *36*

Supplemental information

Transcriptomic analysis of the immune response to *in vivo* gene electrotransfer in colorectal cancer

Mariangela De Robertis, Tim Bozic, Iva Santek, Flaviana Marzano, Bostjan Markelc, Domenico Alessandro Silvestris, Apollonia Tullo, Graziano Pesole, Maja Cemazar, and Emanuela Signori

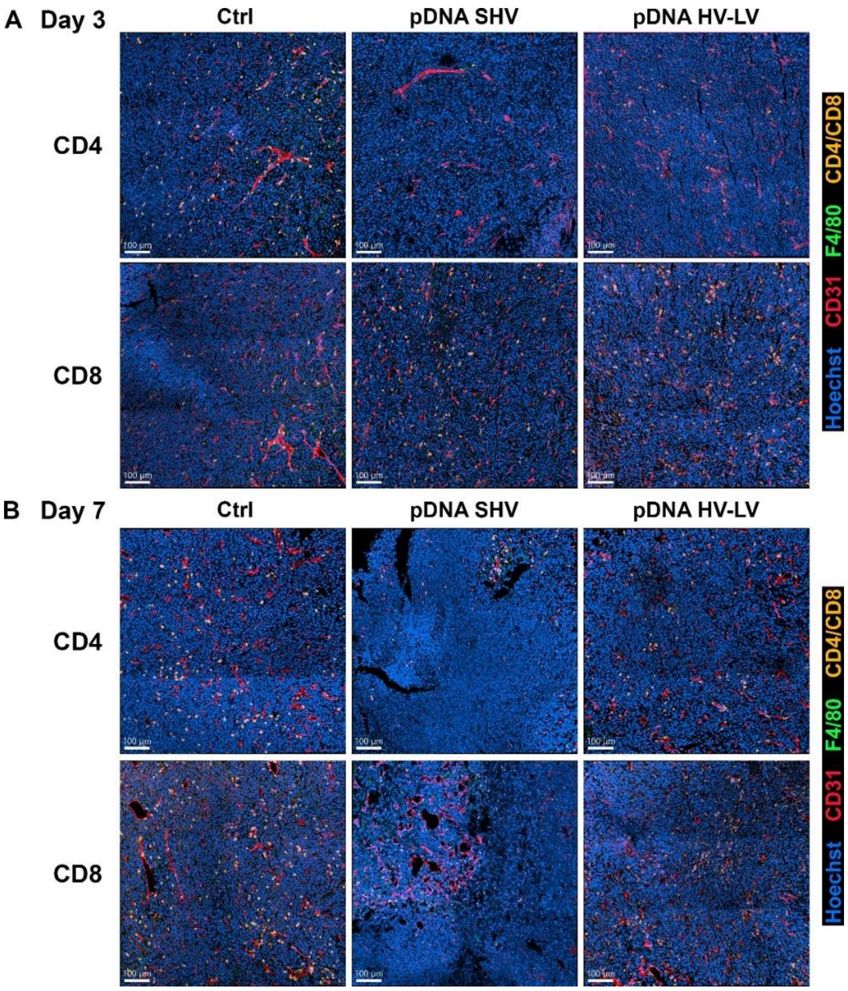

Figure S1. Immune cell infiltration in the tumor centers on day three and day seven after pDNA GET in CT26 murine tumors using SHV or HV-LV pulse protocols. GET was performed using SHV or HV-LV pulses. Tumors were collected on **A)** day three and **B)** day seven after GET for histological analysis. Frozen sections of tumor tissue were stained with anti-CD4 or anti-CD8 (yellow, Cy3), anti-CD31 (red, Alexa 647), anti-F4/80 (green, Alexa 488) and Hoechst 33342 (blue). The representative images of tumor centers are shown. Yellow: Cy3; Red: Alexa 647; Green: Alexa 488; Blue: Hoechst 33342. Scale bar: 100 mm.

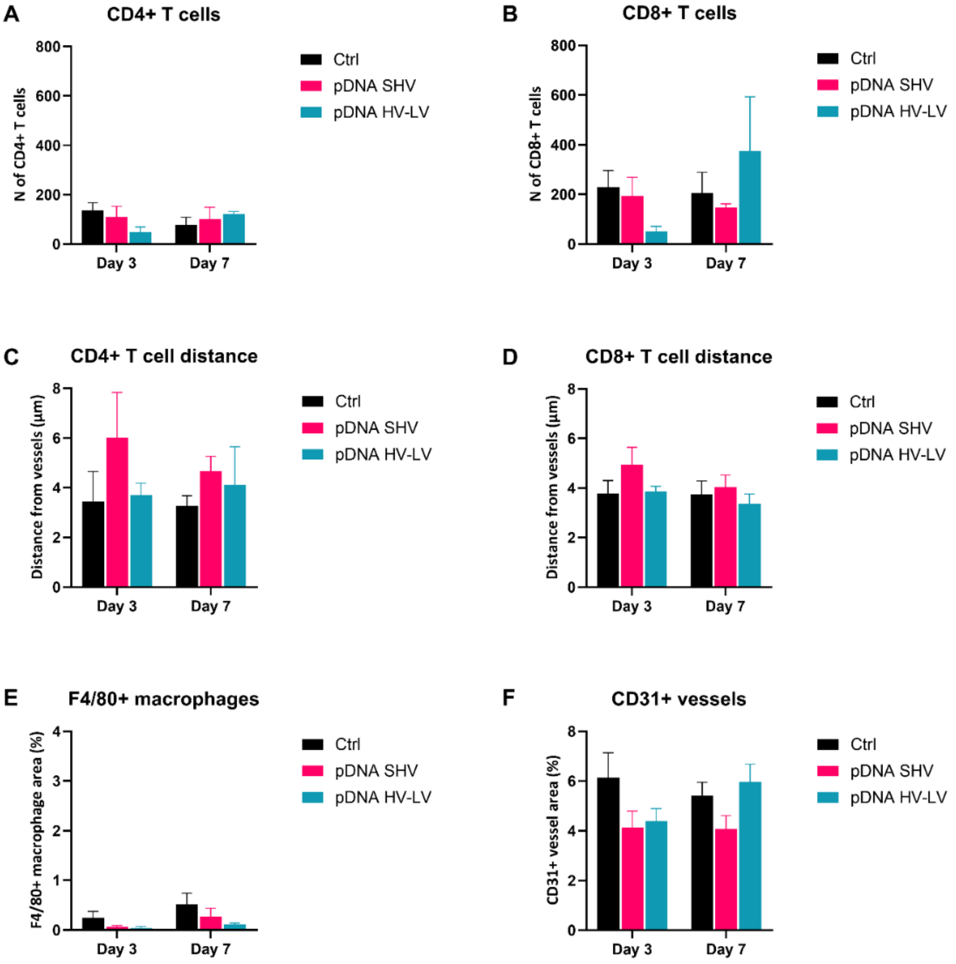

Figure S2. Quantification of immunofluorescence data obtained from images of CT26 tumor centers showed negligible changes in the tumor microenvironment. Graphs represent number of **A)** CD4, **B)** CD8 positive T cells and their **C, D)** distance to tumor vessels, **E)** F4/80 positive macrophages and **F)** the percentage of tumor vessels in CT26 tumors on day 3 and day 7 after GET of pDNA using SHV or HV-LV pulse protocol. Data are presented as AM ± SEM (n ≥ 5). Statistical significance was determined by One-way ANOVA.

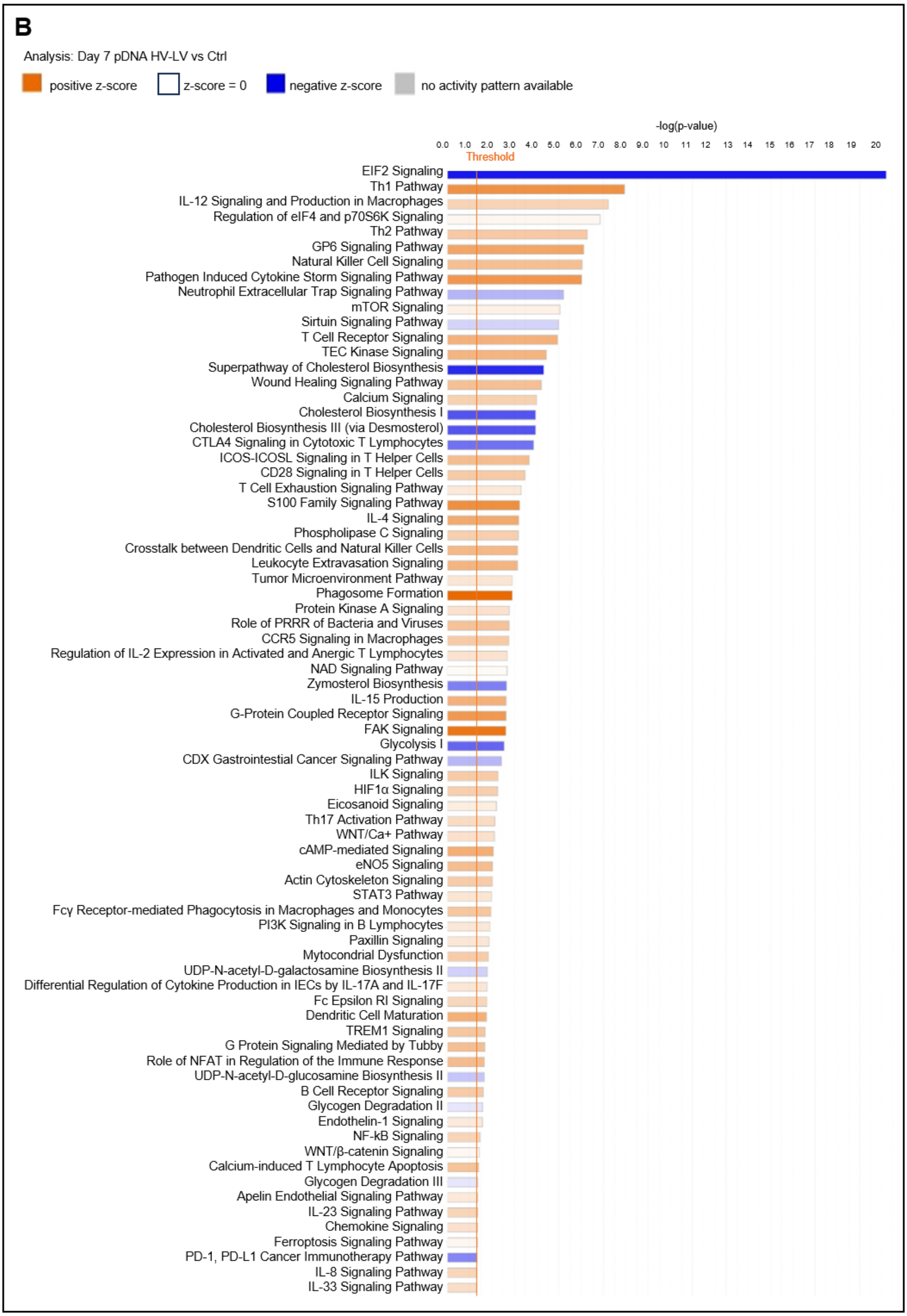

Figure S3. Complete list of the enriched GO canonical pathways in pDNA HV-LV group. Analysis of tumors treated with pDNA GET using H-LV pulse protocol compared to controls at seven days. The plot shows the GO molecular function terms plotted in order of significance.

Table S1. Electric parameters of SHV and HV-LV pulse protocols used for GET of pDNA to CT26 tumors.

Pulse protocol	SHV	HV-LV	
N of pulses	8	4x (1HV + 1LV)	
Electrode distance	6 mm	6 mm	
Voltage/distance [V/cm]	1300	HV: 1300	LV: 150
Duration [ms]	0.1	HV: 0.1	LV: 20
Frequency [Hz]	1	1	
Time between HV and LV [ms]	/	50	
Time between (1HV + 1 LV) [s]	/	1	

Table S2. List of primary and secondary antibodies used for immunofluorescence.

Primary antibodies	Supplier	Cat. No.	Dilution
Anti-CD4 antibody	Abcam	ab183685	1:200
Anti-CD8 antibody	Abcam	ab209775	1:200
Mouse/Rat CD31/PECAM-1 Antibody	R&D systems	AF3628	1:200
F4/80 Monoclonal Antibody (BM8)	ThermoFisher Scientific	14-4801-82	1:200
Secondary antibodies	**Supplier**	**Cat. No.**	**Dilution**
Donkey Anti-Rabbit IgG Antibody (Cy3®)	Jackson Immunoresearch	711-165-152	1:400
Donkey Anti-Goat IgG Antibody (Alexa Fluor® 647)	Jackson Immunoresearch	705-605-147	1:400
Donkey Anti-Rat IgG (Alexa Fluor® 488)	Jackson Immunoresearch	712-545-150	1:400

Table S3. Statistical analysis of the number of CD8+T cells at the tumor edges at day three and day seven after pDNA GET in CT26 murine tumors using SHV or HV-LV pulse protocols.

Ordinary one-way ANOVA (Holm-Šídák)	Mean Diff.	Below threshold?	Summary	Adj. P Value§
Ctrl d3 vs. pDNA Ctrl (SHV) d7	-376.7	Yes	*	0.0438
pDNA Ctrl (SHV) d3 vs. pDNA Ctrl (SHV) d7	-434.5	Yes	*	0.0109
pDNA Ctrl (HV-LV) d3 vs. pDNA Ctrl (SHV) d7	-543.8	Yes	*	0.0109

§Statistical significance was determined by One-way ANOVA. Adj. P-value of < 0.05 was considered to be statistically significant (*P < 0.05 vs non-treated tumors (Ctrl)), between treatments or time points.

Table S4. Statistical analysis of the number of F4/80+ macrophages at the tumor edges at day three and day seven after pDNA GET in CT26 murine tumors using SHV or HV-LV pulse protocols.

Ordinary one-way ANOVA (Holm-Šídák)	Mean Diff.	Below threshold?	Summary	Adj. P Value[§]
Ctrl d3 vs. Ctrl d7	1.465	Yes	***	0.0010
Ctrl d3 vs. pDNA Ctrl (SHV) d3	1.267	Yes	*	0.0159
Ctrl d3 vs. pDNA Ctrl (HV-LV) d7	1.304	Yes	*	0.0136
Ctrl d7 vs. pDNA Ctrl (SHV) d7	-1.029	Yes	**	0.0047
Ctrl d7 vs. pDNA Ctrl (HV-LV) d3	-2.474	Yes	****	<0.0001
pDNA Ctrl (SHV) d3 vs. pDNA Ctrl (HV-LV) d3	-2.275	Yes	****	<0.0001
pDNA Ctrl (SHV) d7 vs. pDNA Ctrl (HV-LV) d3	-1.444	Yes	**	0.0015
pDNA Ctrl (HV-LV) d3 vs. pDNA Ctrl (HV-LV) d7	2.312	Yes	****	<0.0001

[§]Statistical significance was determined by One-way ANOVA. Adj. P-value of < 0.05 was considered to be statistically significant (*P < 0.05, **P < 0.01, ****P < 0.0001 vs non-treated tumors (Ctrl)), between treatments or time points.
